# Synthetic and practical reconstructions of SST and seawater pH using the novel multiproxy SMITE method

**DOI:** 10.1371/journal.pone.0305607

**Published:** 2024-06-25

**Authors:** Hunter P. Hughes, Diane Thompson, Gavin L. Foster, Jonathan Lees, Donna Surge, Christopher D. Standish

**Affiliations:** 1 Department of Earth, Marine and Environmental Sciences, University of North Carolina at Chapel Hill, Chapel Hill, NC, United States of America; 2 Department of Geosciences, University of Arizona, Tucson, AZ, United States of America; 3 School of Ocean and Earth Science, University of Southampton, National Oceanography Centre, Southampton, United Kingdom; Central Marine Fisheries Research Institute, INDIA

## Abstract

Geochemical proxies of sea surface temperature (SST) and seawater pH (pH_sw_) in scleractinian coral skeletons are valuable tools for reconstructing tropical climate variability. However, most coral skeletal SST and pH_sw_ proxies are univariate methods that are limited in their capacity to circumvent non-climate-related variability. Here we present a novel multivariate method for reconstructing SST and pH_sw_ from the geochemistry of coral skeletons. Our Scleractinian Multivariate Isotope and Trace Element (SMITE) method optimizes reconstruction skill by leveraging the covariance across an array of coral elemental and isotopic data with SST and pH_sw_. First, using a synthetic proxy experiment, we find that SMITE SST reconstruction statistics (correlation, accuracy, and precision) are insensitive to noise and variable calibration period lengths relative to Sr/Ca. While SMITE pH_sw_ reconstruction statistics remain relative to *δ*^11^B throughout the same synthetic experiment, the magnitude of the long-term trend in pH_sw_ is progressively lost under conditions of moderate-to-high analytical uncertainty. Next, we apply the SMITE method to an array of seven coral-based geochemical variables (B/Ca, *δ*^11^B, Li/Ca, Mg/Ca, Sr/Ca, U/Ca & Li/Mg) measured from two Bermudan *Porites astreoides* corals. Despite a <3.5 year calibration period, SMITE SST and pH_sw_ estimates exhibit significantly better accuracy, precision, and correlation with their respective climate targets than the best single- and dual-proxy estimators. Furthermore, SMITE model parameters are highly reproducible between the two coral cores, indicating great potential for fossil applications (when preservation is high). The results shown here indicate that the SMITE method can outperform the most common coral-based SST and pH_sw_ reconstructions methods to date, particularly in datasets with a large variety of geochemical variables. We therefore provide a list of recommendations and procedures for users to begin implementing the SMITE method as well as an open-source software package to facilitate dissemination of the SMITE method.

## 1 Introduction

Tropical sea surface temperature (SST) is a critical component of the climate system, linked to a multitude of key variables such as atmospheric moisture content and temperature, cloud cover, and patterns of atmospheric circulation [1]. However, the evolution of tropical SST prior to and during the industrial era remains uncertain due to poor data coverage and changing measurement technology [2]. This uncertainty has significant consequences for attempts to establish a baseline for present-day climate change, impacting our abilities to meet internationally agreed-upon climate targets [3]. Towards this end, massive reef-building (scleractinian) corals have been extensively utilized to reconstruct variations in SST throughout the Holocene [4], thereby filling a critical gap in our knowledge with respect to tropical SST variability.

Over the past several decades, numerous geochemical tracers in scleractinian coral skeletons have been calibrated to local environmental parameters [5], which in turn have been used to reconstruct regional climate variability from seasonal to centennial timescales [6]. Under ideal conditions, a single coral geochemical proxy is largely a function of the climate target of interest, with minimal influence from other variables such as coral growth variations, analytical error, or age model uncertainty. When these conditions are met, coral skeletal geochemistry can be used to robustly reconstruct climate targets such as SST, sea surface salinity (SSS), and seawater pH (pH_sw_), often at monthly resolution [7–9].

The degree of accuracy, precision, correlation, and reproducibility in any coral-based climate reconstruction is often a function of many factors, including (but not limited to): how much natural variability the climate target of interest exhibits in a given location; the uncertainty associated with the chosen observations of the climate target (e.g., *in situ* SST observations versus those derived from remote sensing products); the concentrations of the geochemical proxy in the coral skeleton; how sensitive the geochemical proxy is to the climate target; whether this sensitivity is consistent, both between coral colonies as well as throughout the skeleton of an individual colony; the degree to which other internal or external processes create interference in the relationship between the proxy and the climate target; and whether the age model error is reasonable within the resolution and length of the climate reconstruction.

For example, skeletal strontium-to-calcium ratios (Sr/Ca) are the most commonly used coral-based paleothermometer to date [10, 11]. SST exhibits strong annual cycles even in tropical oceans (∼2°C, and while *in situ* observations of SST in the tropical oceans can be scarce, remote sensing products are becoming increasingly better at capturing SST variations on coral reefs [12], thereby reducing uncertainty with respect to the climate target. Despite a relatively low temperature sensitivity [13, ∼ 0.06 mmol mol^−1^°C^−1^], the high degree of analytical precision for measuring Sr/Ca using inductively coupled plasma optical emission spectrometry [14, ICP-OES] often yields high precision Sr/Ca-based SST estimates (e.g. ± 0.16°C, 1*σ*). However, analytical uncertainty for Sr/Ca increases by an order of magnitude when using inductively coupled plasma mass spectrometry (ICP-MS), particularly when coupled with laser ablation [15]. This is but one contributing factor to the high degree of uncertainty around the regression coefficients for the Sr/Ca—SST relationship, which often vary between taxa, regions, regression techniques, and even different laboratories using similar analytical equipment [13, 16–20]. Uncertainty surrounding the Sr/Ca—SST relationship have also been attributed to biological controls on Sr uptake into the coral aragonite crystal lattice [21–30]. Recent work also suggests that nutrient conditions can create substantial non-SST-related variability in coral Sr/Ca ratios [31]. While much work has gone into improving the reproducibility of coral Sr/Caderived SST estimates [20, 32–36], the wide distribution of coral Sr/Ca regression coefficients often necessitates colony-specific Sr/Ca—SST calibrations, making applications of coral Sr/Ca paleothermometry to fossil coral material challenging at the present time [37, 38].

In the last decade, calibrations using multiple temperature-sensitive proxies, or multiproxy calibrations, have been developed specifically to minimize or even circumvent non-SST-related variability [39–44]. These multiproxy paleothermometers often produce regression coefficients that are more consistent across corals from different regions and taxa [42, 43, 45]. However, this reproducibility comes at the expense of sampling resolution [42] or reconstruction skill relative to the best single-proxy estimator from any given location [43–45]. Furthermore, these multiproxy calibration methods have exclusively focused on SST, leaving other important climate targets such as pH_sw_ untested [46, 47]. This is especially relevant, as the primary coral geochemical proxy for pH_sw_ is its boron isotopic composition (*δ*^11^B). However, it is becoming increasingly apparent that coral skeletal *δ*^11^B ratios are a function of SST, SSS, and the pH of the coral calcifying fluid (pH_cf_), which is upregulated by the coral organism and exhibits increased seasonal variance relative to the pH_sw_ [7, 46–48].

This study presents a novel multiproxy method for reconstructing SST and pH_sw_ across an array of coral geochemical variables. Utilizing the generalized inverse solution [49, 50], our novel Scleractinian Multivariate Isotope and Trace Element (SMITE) method leverages covariance across multiple coral variables to improve the correlation, accuracy, and precision of the reconstruction (henceforth referred to as ‘reconstruction skill’) while yielding reproducible and mechanistically sensible regression coefficients. We first explain how the SMITE method applies the generalized inverse solution to calibrate coral geochemical data (or any coral variable) to SST and pH_sw_. Next, we utilize a synthetic proxy dataset (Sr/Ca, *δ*^18^O, and *δ*^11^B) where the climate targets used to create each ‘pseudoproxy’ are known (SST, SSS, pH_sw_) to explore the effects of analytical noise (random and autocorrelated) and calibration period length on both SMITE SST and pH_sw_ reconstructions. Then, as proof-of-concept, we apply the SMITE method to an array of seven coral variables (B/Ca, *δ*^11^B, Li/Ca, Mg/Ca, Sr/Ca, U/Ca & Li/Mg) measured on two *Porites astreoides* corals collected from the northern fringing reef of Bermuda. These six unique geochemical variables (with the addition of Li/Mg) were chosen to capture a wide array of potential temperature and pH_sw_ dependencies to maximize covariance between the coral variable field and the reconstruction targets. We then examine two ways in which users can exercise control on SMITE reconstruction skill: through selective exclusion of coral variables in the SMITE method, and through a regularization technique that helps constrain SMITE model parameters with minimal impact on reconstruction skill. Finally, based on these findings, we provide a list of recommendations for new SMITE users while suggesting key areas where its use would best improve coral-based climate reconstructions.

## 2 Materials and methods

### 2.1 SMITE method theory and derivation

Multivariate estimations of any climate target from an array of coral variables (e.g., Sr/Ca, *δ*^11^B, linear extension rate) can be represented as a classic inverse problem:

Ax=b (1)

Where *A* is a matrix of age-modeled coral variables, with *p* columns denoting each unique variable and *t* rows denoting time. Vector *b* is of length *t* denoting the climate target across time; and *x* is a vector of length *p* denoting the model parameters that relate *A* to *b*. Thus, *x* contains the regression coefficients that correspond to each coral variable in the *A* matrix and its relationship to *b*. Ideally, the matrix-vector product, or dot-product, of *A* and *x* would yield a perfect estimate of the climate target of interest, *b*.

If *A* has an inverse (*A′*), then *x* could easily be solved as *A′b*. Since only square matrices have inverses, one approach is to calculate the inverse of the square *p* by *p* correlation matrix of *A*, assign *b* as a vector of length *p* containing the correlation coefficients of each coral variable to the climate target, and then solve. However, coral variable data are often highly collinear, and so the model parameters (i.e., the regression coefficients contained in *x*) estimated by this approach would be highly unstable and non-reproducible across space and time.

To bypass the issue of collinearity, the SMITE method utilizes the generalized inverse, or pseudoinverse, solution [49, 50] to calculate model parameters for SST and pH_sw_ estimates from a non-specific array of coral variables. It begins by performing a singular value decomposition [51, 52] on the coral variable field.

$$A_{a}=USV^{T}$$ (2)

Where *A_a_* is a matrix of age-modeled z-score normalized (*μ* = 0, *σ* = 1) coral variables, with *p* columns denoting each variable and *t* rows denoting time. According to the SVD, *A_a_* can be decomposed into three orthogonal matrices: *U*, *S*, and *V*, where *U* and *V* contain basis vectors spanning the data space and the column space, respectively. They are thus column-orthogonal to each other, and each contain the loadings or eigenvectors of the coral variable field. The diagonal matrix *S* contains the singular values of the coral variable field in descending order, representing the variance explained by each eigenvector (or percent variance explained, when normalized by the sum of the diagonal). The SVD and the eigendecomposition, a perhaps more familiar technique that is often used during Empirical Orthogonal Function analysis [53], yield identical results when applied to the covariance matrix of a particular data field. In this case, the eigenvalues calculated from the eigendecomposition are identical to the singular values calculated from the SVD, and the eigenvectors are identical to the *U* and *V* matrices. However, the SVD offers a number of additional benefits over the eigendecomposition, one of which is that it can be applied to non-square matrices [54]. Therefore, all information between coral variables is retained when calculating SMITE model parameters utilizing the SVD.

As mentioned before, *A′* cannot be determined because *A* is not square, and calculating the inverse of the covariance matrix of *A* creates collinearity and violates one of the basic assumptions of regression (independent predictor variables). However, we have shown that the z-score normalized coral variable field matrix *A_a_* can be decomposed into three orthogonal matrices, for which an inverse does exist. Thus, a generalized inverse, or ‘pseudoinverse’, of the *A* matrix (*A*_†_) can be calculated by rearranging the terms of the decomposed *A* matrix.

$$A_{†}=VS^{-1}U^{T}$$ (3)

Consequently, a pseudoinverse solution [55] can be calculated from the dot-product of *A*_†_ and *b*.

$$x_{†}=A_{†}b=VS^{-1}U^{T}b$$ (4)

Where *x*_†_ is a vector of length *p* containing the SMITE model parameters for each unique coral variable. Henceforth, we will simply refer to the *x*_†_ vector as SMITE model parameters. We will refer to the specific value of each SMITE model parameter as its ‘*x*_†_ value’. Thus, the dot-product of the z-score normalized coral variable field matrix and SMITE model parameters yield normalized estimates of the climate target of interest.

$$A_{a}x_{†}={\hat{b}}_{a}$$ (5)

These can then easily be converted to absolute estimates using the standard deviation (*σ*) and mean (*μ*) of the climate target in the calibration dataset.

$$\hat{b}=\left({\hat{b}}_{a}*\sigma_{b}\right)+\mu_{b}$$ (6)

Importantly, the pseudoinversion of the *A* matrix and the calculation of *x*_†_ assumes that the columns of *A* exhibit a linear relationship to *b*. In the case of coral SST proxy data, the assumption of linearity often holds within a particular temperature range [56]. However, if nonlinearity is observed in a particular variable’s relationship to the climate target of interest, it may be necessary to transform the coral variable prior to its entry into the *A* matrix so that it exhibits a more linear relationship to the climate target of interest.

Prior to calculating SMITE model parameters in Eq 4, higher order singular values (i.e., ones describing less variance in the dataset) and their corresponding basis vectors in *U* and *V* can be ‘truncated’ from the pseudoinverse of the coral variable field.

$${A_{†}}_{T}=V_{T}S_{T}^{-1}U_{T}^{T}b_{a}$$ (7)

Where subscript *T* denotes that the corresponding matrix has had certain columns removed or truncated. This is a common regularization technique used in Principal Components Regression [57]. The accuracy, precision, correlation, and reproducibility of any SMITE reconstruction can vary depending on the level of regularization implemented (see sections 3.2.2 and 4.2). Ideally, this step is taken to remove singular values that are close to zero and prevent the SMITE method from overfitting the model to random noise and or variability not related to the climate target of interest. We provide guidance for choosing the optimum level of regularization in section 3.

**Fig 1:**
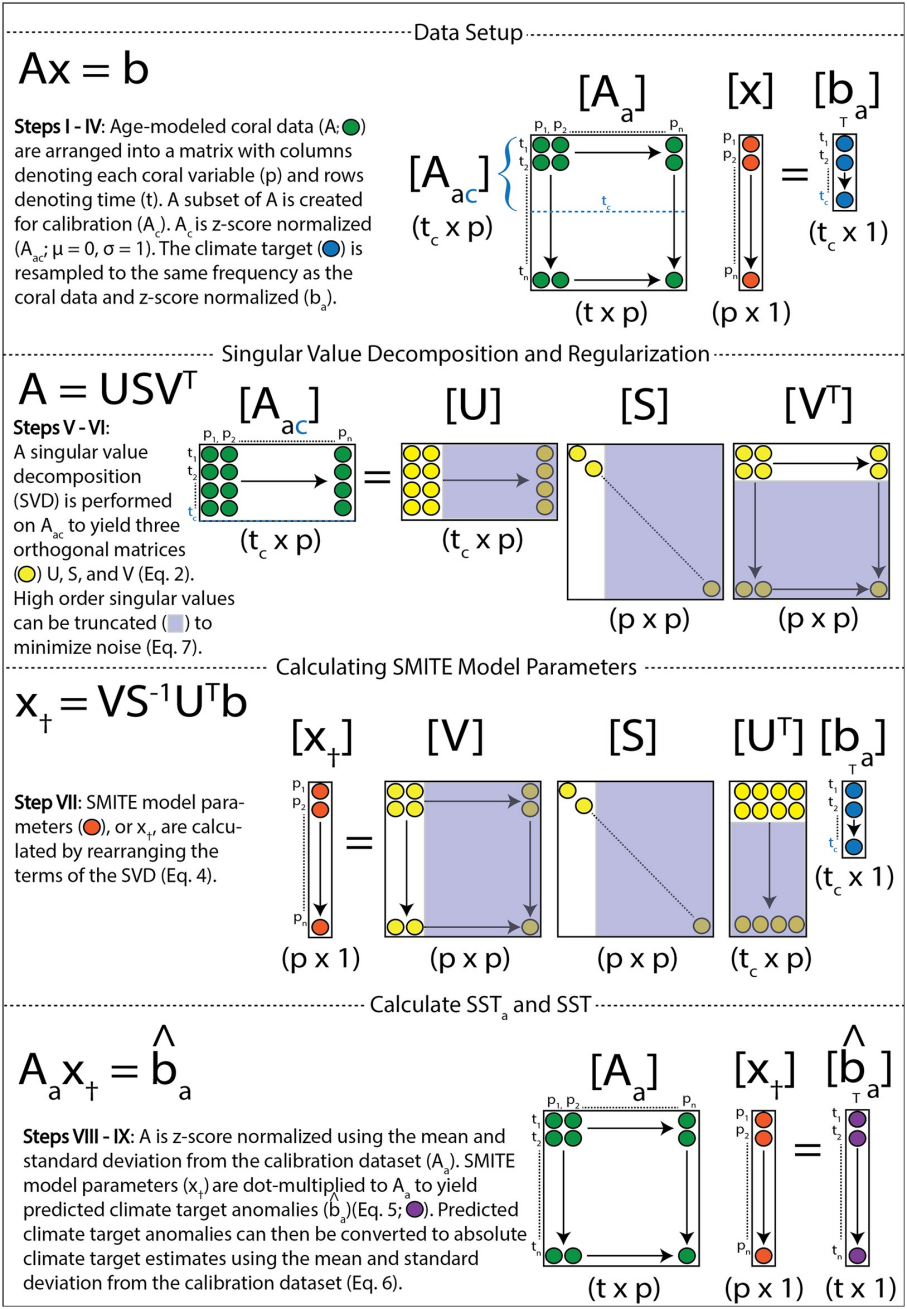
Procedural diagram for implementing the SMITE method on a coral variable dataset.

The procedure for reconstructing SST or pH_sw_ from an array of coral variables using the SMITE method is listed below, along with a procedural diagram (Fig 1).

1. Age-modeled coral variables are arranged into a matrix with columns denoting each coral variable and rows denoting time (*A*).
2. A subset of *A* is created for calibration (*A_c_*).
3. *A_c_* is z-score normalized (*A_ac_*; mean-subtracted, divided by the standard deviation).
4. SST is (if necessary) resampled to the same frequency as the coral variable data and z-score normalized (*b_a_*).
5. An SVD is performed on *A_ac_* to yield three orthogonal matrices, *U*, *S*, and *V* (Eq 2).
6. (OPTIONAL) The *U*, *S*, and *V* matrices are truncated to remove higher order singular values (Eq 7).
7. SMITE model parameters (*x*_†_) are calculated by rearranging the terms of the SVD (Eq 4).
8. *A_ac_* and *x*_†_ are dot-multiplied to yield predicted climate target anomalies during the calibration period (${\hat{b}}_{a}$)(Eq 5). To yield predicted climate anomalies for the entire length of the record, *A* must be z-score normalized using the mean and standard deviation of the calibration dataset (*A_a_*) before it is dot-multiplied to *x*_†_.
9. Predicted climate target anomalies are converted to absolute climate target estimates ($\hat{b}$) using the mean and standard deviation from the calibration dataset (Eq 6).

### 2.2 Synthetic pseudoproxy dataset

**Fig 2:**
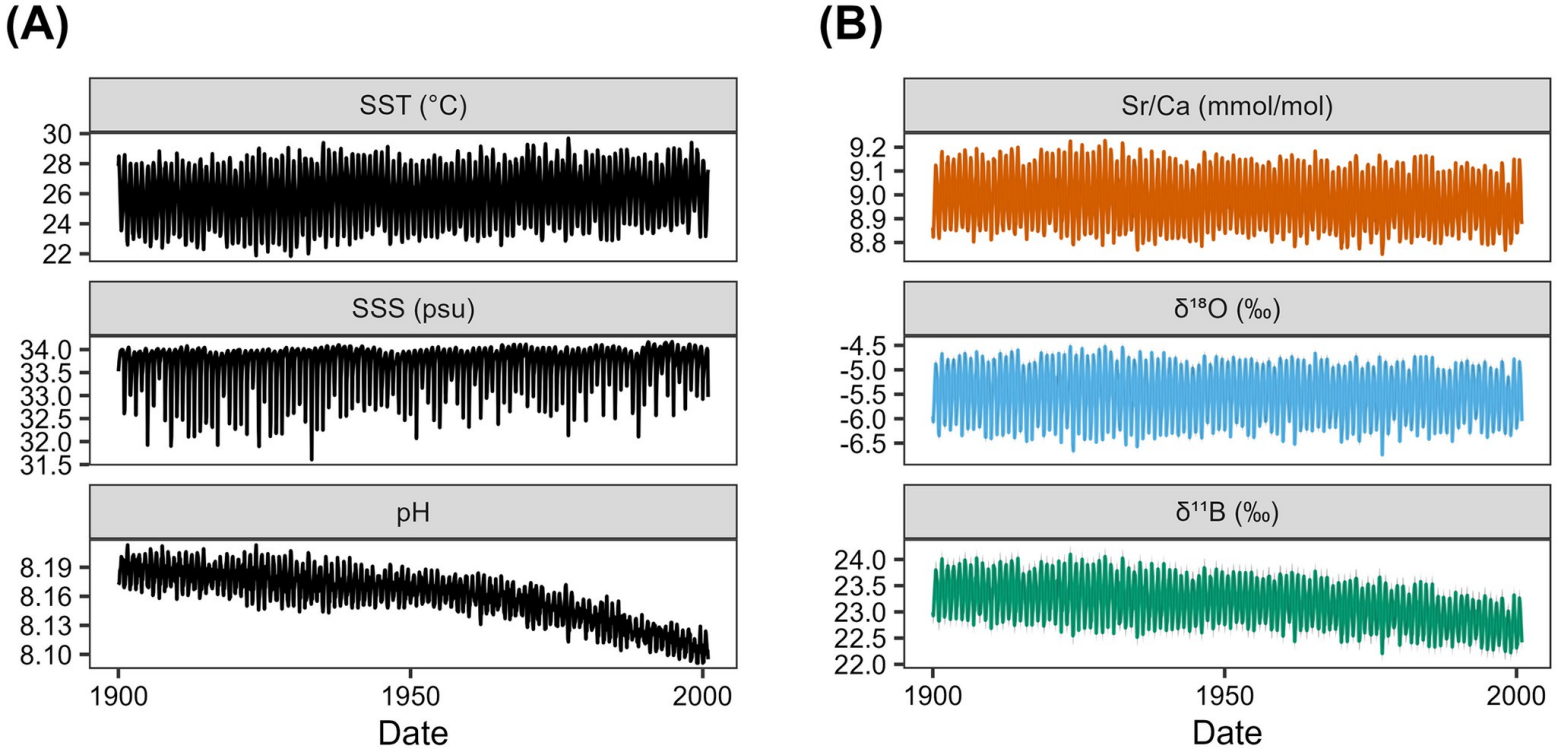
Monthly time series of three synthetic pseudoproxies (B) calculated from environmental information spanning the 20th century off the east coast of Australia (A) [62, 63]. The gray shaded region in each panel, which is barely visible, indicates the minimum (analytical) uncertainty associated with each pseudoproxy. All three pseudoproxies exhibit an idealized relationship with their corresponding environmental variable(s) of interest (Sr/Ca ∼ SST; *δ*^18^O ∼ SST + SSS; *δ*^11^B ∼ SST + SSS + pH_sw_).

We created three synthetic ‘pseudoproxies’ (Sr/Ca, *δ*^18^O, *δ*^11^B) with various amounts of environmental information encoded into each, based on their theoretical dependence on SST, SSS and pH_sw_ (Fig 2). Because we created this dataset with three coral variables and three potential climate targets, this is considered a square system (i.e., the number of predictor variables equals the number of unknown variables), which is important when considering regularization (see sections 3.2.2 and 4.2). The means by which we calculated these three synthetic pseudoproxies are highly idealized, meaning that each pseudoproxy has a near-perfect relationship with its corresponding climate target(s). However, the uncertainty in each proxy’s relationship to the climate target is considered in our experimental design, which examines how uncertainty in reconstructed SST and pH_sw_ estimates increases as the degree of Gaussian and autocorrelated noise increases. The magnitude of Gaussian noise and autocorrelation considered in our experiment (Table 1) is beyond the typical range observed in most coral-based paleoclimate studies [16, 58, 59]. We expect therefore that all linear sources of uncertainty, and their subsequent impacts on SST and pH_sw_ estimates, are accounted for in this conservative analysis. The minimum uncertainty for each synthetic pseudoproxy was taken from the literature as analytical uncertainty. For synthetic Sr/Ca values, this was taken to be 0.009 mmol/mol, or approximately 0.1% RSD [14]. For synthetic *δ*^18^O values, we used an analytical uncertainty of 0.1‰ [60]. For synthetic *δ*^11^B values, analytical uncertainty was taken to be 0.09‰ [61].

**Table 1:** Mean (*μ*), standard deviation (*σ*), range of analytical errors (*ϵ*), and correlation coefficients (r) for all synthetic proxies to each environmental variable.

Synthetic Proxy	*μ*	*σ*	*ϵ*_min_ − *ϵ*_max_ (RSD)	r − SST	r − SSS	r − pH_sw_
**Sr/Ca (mmol/mol)**	8.98	0.12	0.009–0.180 (0.1–1.9%)	−1.00	0.68	0.54
***δ*^18^O (‰)**	−5.49	0.51	0.10–0.21 (1.8–3.7%)	−0.98	0.80	0.50
***δ*^11^B (‰)**	22.90	0.66	0.18–0.62 (0.78–2.68%)	−0.92	0.63	0.82

Monthly SST and pH_sw_ data from the Great Barrier Reef (18.5°S, 149.5°E) between 1900 and 2000 were acquired from Lenton et al. (2016) [62], a 20th century reconstruction of SST, SSS and pH_sw_ across the Great Barrier Reef (n = 1212). SSTs ranged from 21.83°C to 29.69°C (*μ* = 25.92°C ± 1.91, 1*σ*), with a minor but significant warming trend of 0.08°C per decade (*p* < 0.0001). Seawater pH ranged from 8.09 to 8.21 (*μ* = 8.16 ± 0.03) and exhibits two significant negative trends pre-1950 (0.004 units per decade) and post-1950 (0.014 units per decade). Although SSS data are also available from this dataset for the same time interval, these data simply repeat the same annual cycle of SSS throughout the 20th century with no interannual or decadal variability. To better reproduce long-term changes in SSS, we used SSS data from the ORA20C dataset [63] from the same location and time interval. This dataset extends back through the 20th century and is an advanced data assimilation product that tunes the output of the European Center for Medium-range Weather Forecasts twentieth century reanalysis, ERA-20C, to *in situ* observations. SSS variations from this location in the ORA20C dataset exhibit a highly skewed left distribution (*μ* = 33.69 ± 0.43 psu), with values ranging from 31.61 to 34.17 due to episodic freshwater runoff events.

According to hindcast archived data from the CSIRO Environmental Modelling Suite implemented by the Australian Institute of Marine Science (https://research.csiro.au/cem/software/ems/), SSS in this region between 2010 and 2022 ranged from 34.7 to 35.6 psu and exhibit a slightly skewed left distribution (*μ* = 35.23 ± 0.16 psu). We acknowledge that the distributions of ORA20C and CSIRO SSS are statistically distinct from one another, both in terms of mean and variance (*p* < 0.001). However, the purpose of including SSS in the synthetic experiment is to create interference in both synthetic *δ*^18^O and *δ*^11^B values for reconstructing SST and pH_sw_, respectively. Thus, there are two important aspects of SSS that we wish to reproduce for the purposes of this experiment: long-term variability (interannual to decadal), and the covariance between SSS and SST. The ORA20C SSS dataset for this region exhibits substantial interannual and decadal-scale variability, while the Lenton et al. (2016) SSS dataset exhibits none. With respect to covariance, the CSIRO dataset shows that SST and SSS are moderately anti-correlated in this region (r = −0.58). SST and SSS data from Lenton et al. (2016) exhibit a slightly weaker anti-correlation (r = −0.53), while SST data from Lenton et al. (2016) and ORA20C SSS exhibit a slightly stronger anti-correlation (r = −0.68). Since ORA20C SSS exhibits both long-term variability and similar covariance to SST as the observed CSIRO data, we chose to use the ORA20C SSS dataset for our synthetic experiment.

Synthetic Sr/Ca ratios were calculated as a function of SST using the mean slopes and intercepts for the Sr/Ca ∼ SST relationship from Corrège (2006) [13].

$$Sr/Ca_{c}=-0.0607\left(0.0090\right)SST+10.553\left(0.292\right)SSS$$ (8)

Where *SST* is sea surface temperature in degrees Celsius. Synthetic Sr/Ca (*Sr/Ca_c_*) ratios ranged from 8.75 to 9.23 mmol/mol (*μ* = 8.98 mmol/mol ± 0.12).

Synthetic *δ*^18^O values were calculated as a function of both SST and SSS using equation 1 from Thompson et al. (2011) [64].

$$\delta^{18}O_{c}=-0.22SST+0.27SSS$$ (9)

The regression slopes for SST and SSS were chosen using the same criteria from Thompson et al. (2011). The SST slope is the organic slope of the *δ*^18^O and SST relationship, while the SSS slope is based on basin-scale seawater *δ*^18^O and SSS regression estimates [65]. Synthetic *δ*^18^O values ranged from −6.74 to −4.52‰(*μ* = −5.49 ± 0.51‰).

Synthetic *δ*^11^B values were calculated as a function of SST, SSS and pH_cf_. They were determined by rearranging the pH-dependent equation from Zeebe and Wolf-Gladrow (2001) [48] to solve for the boron isotope ratio of carbonate (*δ^11^B_c_*).

$$\delta^{11}B_{c}=pK_{B}-log\left(\frac{\delta^{11}B_{sw}-\delta^{11}B_{c}}{\delta^{11}B_{c}-\delta^{11}B_{sw}+1000\left(\alpha -1\right)}\right)$$ (10)

Where the boron isotope ratio of seawater (*δ^11^B_sw_*) is 39.61‰ [66], and the mass fraction factor between boric acid and borate ion (*α*) is 1.0272 [67]. The negative log of the dissociation constant between boric acid and borate ion (*pK_B_*) is a function of both temperature and salinity [68]. We therefore calculated *pK_B_* at each time interval by taking the negative log of the *K_B_* equation from Dickson (1990) [68]. The values of *pK_B_* ranged between 8.56 and 8.64 given a temperature range between 21.83 and 29.69°C and a salinity range between 31.61 and 34.17 psu.

These calculations yield synthetic *δ*^11^B values between 18.30 and 19.64‰, which is expected given the pH of seawater. However, corals upregulate their internal pH relative to seawater [46] while also often exhibiting increased seasonal variance [69]. Thus, to yield synthetic *δ*^11^B values consistent with those observed in coral aragonite, we calculated pH_cf_ from pH_sw_ using equation 13 from D’Olivo et al. (2019) [7].

$$pH_{cf}=0.49pH_{sw}+4.93-0.02SST$$ (11)

Note that the temperature sensitivity of synthetic *δ*^11^B values is realized in its dependence on both *pK_B_* as well as pH_cf_ as specified in Eq 11. Meanwhile, the salinity sensitivity of synthetic *δ*^11^B values is only realized in its dependence on *pK_B_*. Synthetic *δ*^11^B values ranged from 22.21—24.10‰ (*μ* = 23.17‰± 0.41).

### 2.3 *P. astreoides* application study

#### 2.3.1 Coral sampling and sample preparation

**Fig 3:**
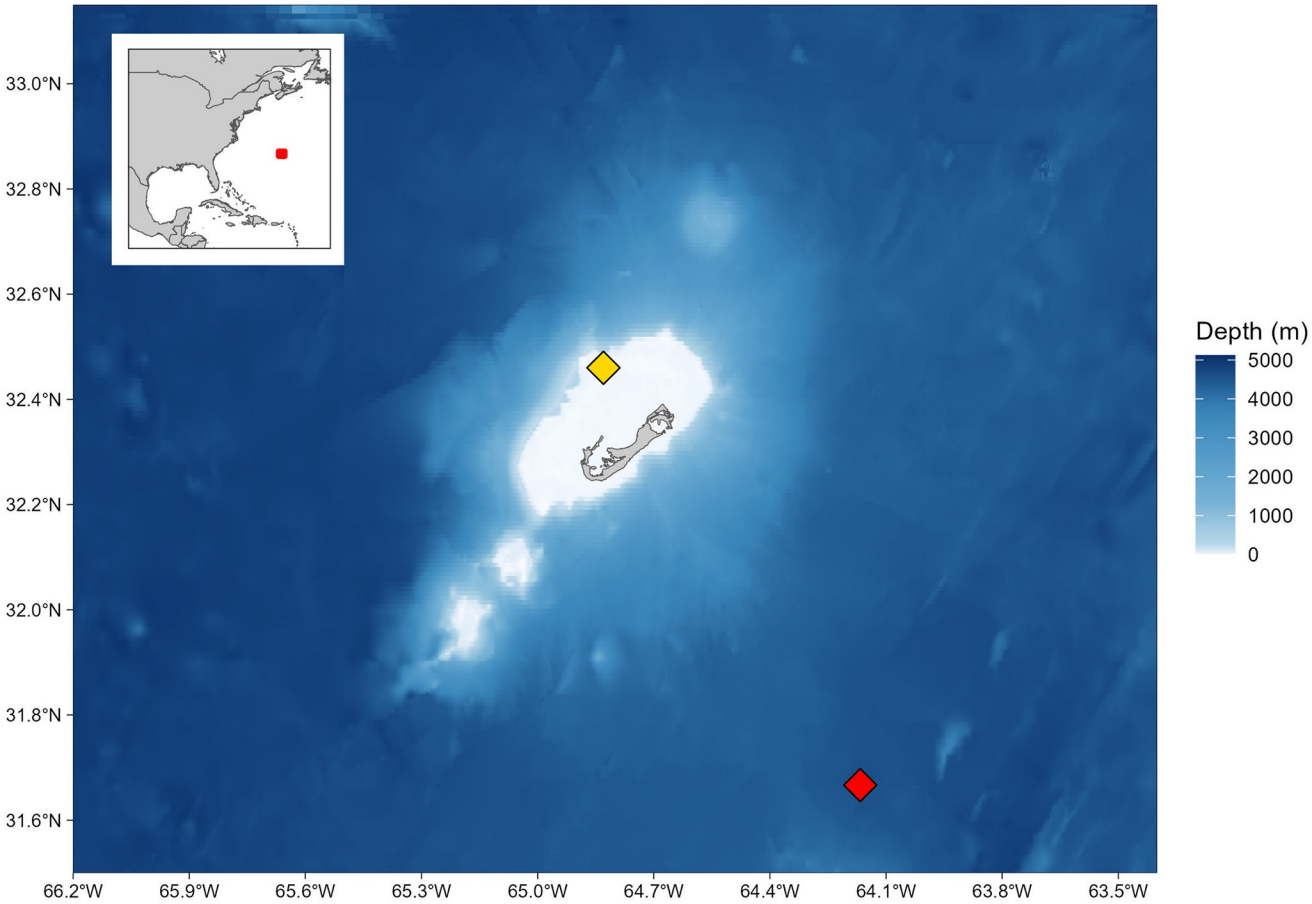
Collection site of *P. astreoides* corals 1B and 3B on the northern fringing reef of Bermuda. The yellow diamond indicates the location of National Oceanographic and Atmospheric Administration’s PMEL MAPCO2 buoy moored at Hog Reef (32.457°N, 64.835°W), meters from where corals 1B and 3B were cored. The red diamond indicates the location of the Bermuda Atlantic Time-series Study (BATS) sampling station (∼32.67°N, 64.17°W. The red box on the inlayed map indicates the borders of the larger map. Bathymetry data displayed was collected from the National Oceanographic and Atmospheric Association via the open-source R package ggOceanMaps [70]. Shoreline data are republished from [71] under a CC BY license, with permission from Dr. Walter H.F. Smith, original copyright 1996.

In November 2014 a 5 cm diameter core was sampled from two *P. astreoides* colonies (1B and 3B, respectively) located on Hog Reef, Bermuda (32.457°N, 64.835°W), at a depth of 10 and 12 m using a 1 horsepower hand-held pneumatic air drill with a custom made 5-by-15 cm diamond core bit (Fig 3). Corals were sampled as close to the National Oceanographic and Atmospheric Administration’s PMEL MAPCO2 buoy moored at Hog Reef as possible (within 5 m). After sampling, voids in the coral skeleton were filled with cement plugs to prevent boring organisms from inhabiting the holes, and to encourage further coral growth. Cores were then transported to the University of Southampton for geochemical analyses. The outer tissue layers of each coral core were removed using a WaterPik before being left to soak in pure ethanol for 5 minutes followed by deionised water. A clean sintered diamond cut-off wheel was used to cut the cores into ∼7 mm thick slabs, both of which were then divided into two sections. Each section was polished using a rotary diamond grinder followed by silicon carbide grinding paper down to 4 *μ*m. Samples were cleaned over two days in a solution of 20% H_2_O_2_ and 2.0 M NH_3_ before being rinsed, ultrasonicated for 10 minutes, then rinsed again in 18.2 M*Ω* (ultrapure) water and left to dry in a flow box. Finally, the topmost sections containing the most recent growth were mounted onto glass slides in preparation for geochemical analyses.

#### 2.3.2 Geochemical analysis

Geochemical analyses were carried out at the Geochemistry laboratory, School of Ocean and Earth Sciences, University of Southampton. Elemental analyses (Li, B, Mg, Ca, Sr, U) were performed on an Agilent (Agilent Technologies Inc., CA, USA) 8900 Triple Quadrupole ICP-MS coupled to an Elemental Scientific Lasers (Bozeman, MT, USA) NWR193 excimer laser ablation system with a TwoVol2 ablation chamber. On-peak blank corrections based on the mean intensities of preceding and succeeding blank measurements were applied offline, as was instrumental drift and mass bias corrections which employed sample-standard bracketing with coral reference material JCp-1 (*Porites sp.* coral) using the values published by Hathorne et al. (2013b) [40]. Samples and standards were ablated in line mode, with sample analyses ablating the same tracks previously used for the *δ*^11^B analyses (see below). Standard analysis consisted of approximately 285 integration cycles of 0.42 s. Samples were analyzed along 30 parallel and adjoining tracks amounting to ∼6 mm long transects that each consisted of ∼1400 integration cycles of 0.42 s. Operating conditions are detailed in Table 2. Reference material PS69/318–1, a cold-water calcitic scleraxonian octocoral coral, was ablated throughout the analytical session as a guide to accuracy, internal precision, and external reproducibility (n = 10). Internal precision, expressed as 2 standard errors (SE) of the mean of 1200 integration cycles, was ≤7% for B/Ca, Mg/Ca, and Sr/ Ca, and ≤16% for Li/Ca, U/Ca and Li/Mg. External reproducibility, expressed as 2*σ* of the mean of the 10 analyses, was <6% for B/Ca, Mg/Ca, Sr/ Ca and Ba/Ca, 12% for Li/Ca and Li/Mg, and 29% for U/Ca. Mean accuracy was within 10% of solution values for all ratios except Li/Mg which was within 11% [72].

**Table 2:** Typical operating conditions for laser ablation ICP-MS analysis.

	*δ*^11^B isotope analysis	Trace element analysis
**Instrument**		
Mass Spectrometer	Thermo Scientific Neptune Plus multi-collector inductively coupled plasma mass spectrometer	Agilent 8900 Triple Quadrupole inductively coupled plasma mass spectrometer
Laser Ablation System	Elemental Scientific Lasers NWR193 excimer laser ablation system with a TwoVol2 ablation chamber	Elemental Scientific Lasers NWR193 excimer laser ablation system with a TwoVol2 ablation chamber
RF Power	1400 W	1550 W
Cones	Nickel skimmer (X) and jet sample	Standard nickel sample cone and XT skimmer
**Gas Flows**		
Cooling gas (argon)	16 l min^−1^	13 l min^−1^
Auxiliary gas (argon)	0.7 l min^−1^	0.8 l min^−1^
Make-up gas (argon)	1.0 l min^−1^	0.6 l min^−1^
Ablation cell carrier gas (helium)	0.85–1.00 l min^−1^	0.5 l min^−1^
Additional gas (nitrogen)	0.004–0.007 l min^−1^	0.01 l min^−1^
**Ablation Conditions**		
Laser power density	∼4 J cm^−2^	∼1.8 J cm^−2^
Laser repetition rate	12 Hz	5 Hz
Laser beam size	100–150 *μ*m diameter	150 *μ*m diameter
Laser tracking speed	10 *μ*m s^−1^	10 *μ*m s^−1^
Ablation mode	Line	Line

Boron isotope analyses were performed on a Thermo Scientific Neptune Plus MC-ICP mass spectrometer coupled to an Elemental Scientific Lasers NWR193 excimer laser ablation system with a TwoVol2 ablation chamber, broadly following previously described analytical protocols [59, 73]. Data were collected in static mode, with ^10^B and ^11^B measured on the L3 and H3 Faraday cups, both of which were installed with 10^12^*Ω* resistors. The laser system was again operated in line mode, with standard measurements consisting of 100 integration cycles of 2.194 s (100–120 *μ*m laser beam diameter) and sample measurements consisting of ∼282 integration cycles of 2.194 s (150 *μ*m laser beam diameter). Prior to data collection, standards and samples were ablated to remove any surface contamination (laser power density of ∼2 J cm^-2^, laser repetition rate of 10 Hz, laser tracking speed of 200 *μ*m s^-1^). Dynamic blank corrections were applied cycle by cycle assuming a linear relationship between preceding and succeeding blank measurements (each consisting of 22 integration cycles of 2.194 s); instrumental mass bias was corrected by sample-standard bracketing with glass reference material NIST SRM610 and the isotope composition published by le Roux et al. (2004) [74] and Standish et al. (2019) [59]; and matrix interferences from scattered ions [59] were corrected based on the power-relationship between *δ*^11^B inaccuracy and ^11^B/Ca_interference_ for pressed pellets of carbonate reference materials JCp-1 and JCt-1 (*Tridacna gigas*), and where Ca_interference_ was measured at m/z of 10.10 on the L2 Faraday cup. All corrections were applied offline. Standard data were screened, with cycles falling outside 2*σ* of the mean removed. Internal reference material PS69/318–1 was ablated throughout the analytical session as a guide to internal precision, external reproducibility, and accuracy. Internal precision, expressed as 2SE of the mean of the 100 integration cycles, was ≤0.4‰. The mean *δ*^11^B of the repeat analysis (n = 18) was 13.61 ± 0.54‰(2*σ*), consistent with a solution measurement of 13.83 ± 0.29‰ [59].

#### 2.3.3 Age model and environmental reconstructions

For each coral we combined 10 adjoining and parallel laser transects into a single time series for each geochemical variable by averaging data in depth space. We report the uncertainty on the mean for each measurement as the SE of the 10 parallel laser transects. An age model was then constructed based firstly upon consideration of an x-ray image showing density banding and the date of collection. This was then refined by tuning annual variations in Sr/Ca to SST, where minima, maxima, and mid-points were considered contemporaneous with SST maxima, minima, and mid-points, respectively. Using this method, we calculate that data from coral 1B ranges from June 2010 to April 2013, and data from coral 3B ranges from June 2010 to September 2013.

We created an *in situ* SST and pH_sw_ dataset ranging throughout the study period (May 2010 to September 2013) by combining two independent *in situ* environmental datasets from the northern fringing reef of Bermuda. The first dataset (from January 2011 to September 2013) was acquired from the National Oceanographic and Atmospheric Administration PMEL MAPCO2 buoy moored at Hog Reef (32.457°N, 64.835°W). Corals 1B and 3B grew several meters away from the Hog Reef Buoy, which has been continuously measuring seawater partial pressure CO_2_, SSS and SST since 2011 with only minor interruptions (https://www.pmel.noaa.gov/co2/story/Hog+Reef). A continuous record of SST and pH_sw_ was calculated from these data by assuming a constant seawater alkalinity of 2357 *μ*mol/kg [75] for most of the study interval (July 2011 to March 2013) and solving the full carbonate system using alkalinity and the partial pressure of CO_2_ using the seacarb package in R [76]. This assumption is justified because both CO_2_ and pH_sw_ are determined by the alkalinity / dissolved inorganic carbon ratio [77], therefore using a range of alkalinity from 2300 to 2400 *μ*mol/L (the range observed by Courtney et al., 2017 [75]) changes our estimate of pH_sw_ by only 0.015 pH units.

To extend our environmental data beyond the interval covered by the Hog Reef Buoy to the bottom of our time series, we utilized a second dataset consisting of shipboard SST and pH_sw_ data acquired from the Bermuda Atlantic Time-series Study [78, BATS; 32.67°N, 64.17°W], which has been measuring SST and pH_sw_ at monthly intervals since 1983. Due to both the discontinuous nature of the BATS data, and the lack of Hog Reef data prior to 2011, we established a linear model between BATS and Hog Reef SST (n = 41, r = 0.78, *p* < 0.001) as well as BATS and Hog Reef pH_sw_ (n = 41, r = 0.83, *p* < 0.001). We then created an *in situ* SST and pH_sw_ calibration dataset by combining the predicted values of Hog Reef data (when BATS data was available) and observed Hog Reef data (when BATS data was not available).

Finally, we calculate Sr/Ca-derived SST estimates for corals 1B and 3B using ordinary least squares regression, and we calculate *δ*^11^B-derived pH_sw_ using Eqs 10 and 11. To estimate *pK_B_*, we use our combined BATS and Hog Reef *in situ* SST dataset, as well as SSS data from BATS alone. SSS on Hog Reef is relatively invariant, and there is little offset between Hog Reef SSS and BATS SSS. Missing SSS values from the BATS dataset (3 out of 40 months) were linearly interpolated between adjacent months for the purposes of generating a continuous *pK_B_* dataset.

### 2.4 Error assessments

We use three metrics to quantitatively compare SMITE SST and pH_sw_ estimates with those derived from Sr/Ca ratios and *δ*^11^B values, respectively: the correlation coefficient (r), the root-mean squared error (RMSE), and the standard error of prediction (SEP). Each metric provides a measure of the correlation, accuracy, and precision of the reconstruction, respectively. The SEP is defined as the uncertainty in derived SST estimates based on the uncertainty in both the climate target (SST, pH_sw_) as well as the uncertainty in the corresponding coral variable(s). Given that our SST measurements are derived from, or modeled after, temperatures derived from *in situ* loggers, uncertainty for temperature was fixed at 0.02°C (https://www.onsetcomp.com/products/data-loggers/u22-001). Uncertainty for our pH_sw_ measurements were fixed at 0.02 units. Uncertainty for SSS measurements (used in calculating *pK_B_* for *δ*^11^B-derived pH_sw_ estimates) were based on BATS CTD measurements and also fixed at 0.02 psu.

The SEP for each climate target reconstruction is calculated using a bootstrap Monte Carlo approach. At each iteration (i = 1,…,10000), each individual measurement in both the coral variable and climate target fields are randomly resampled from a normal distribution with a mean equal to the given variable/climate target value (*μ*_*i*_) and a standard deviation equal to the specified error (*s*_*i*_). Model parameters are then estimated from the perturbed coral variable and climate target fields, and SST/pH_sw_ estimates for each data point are stored. The 95% confidence interval for each predicted value is determined from the distribution of predicted values derived from each Monte Carlo iteration. The SEP is then determined as the average distance from the mean to the upper and lower bounds of the 95% confidence interval, divided by 1.96 [79]. The 95% confidence interval for the SEP itself is then defined as the standard deviation of the SEP throughout each calibration dataset, multiplied by 1.96.

## 3 Results

### 3.1 Synthetic study

**Fig 4:**
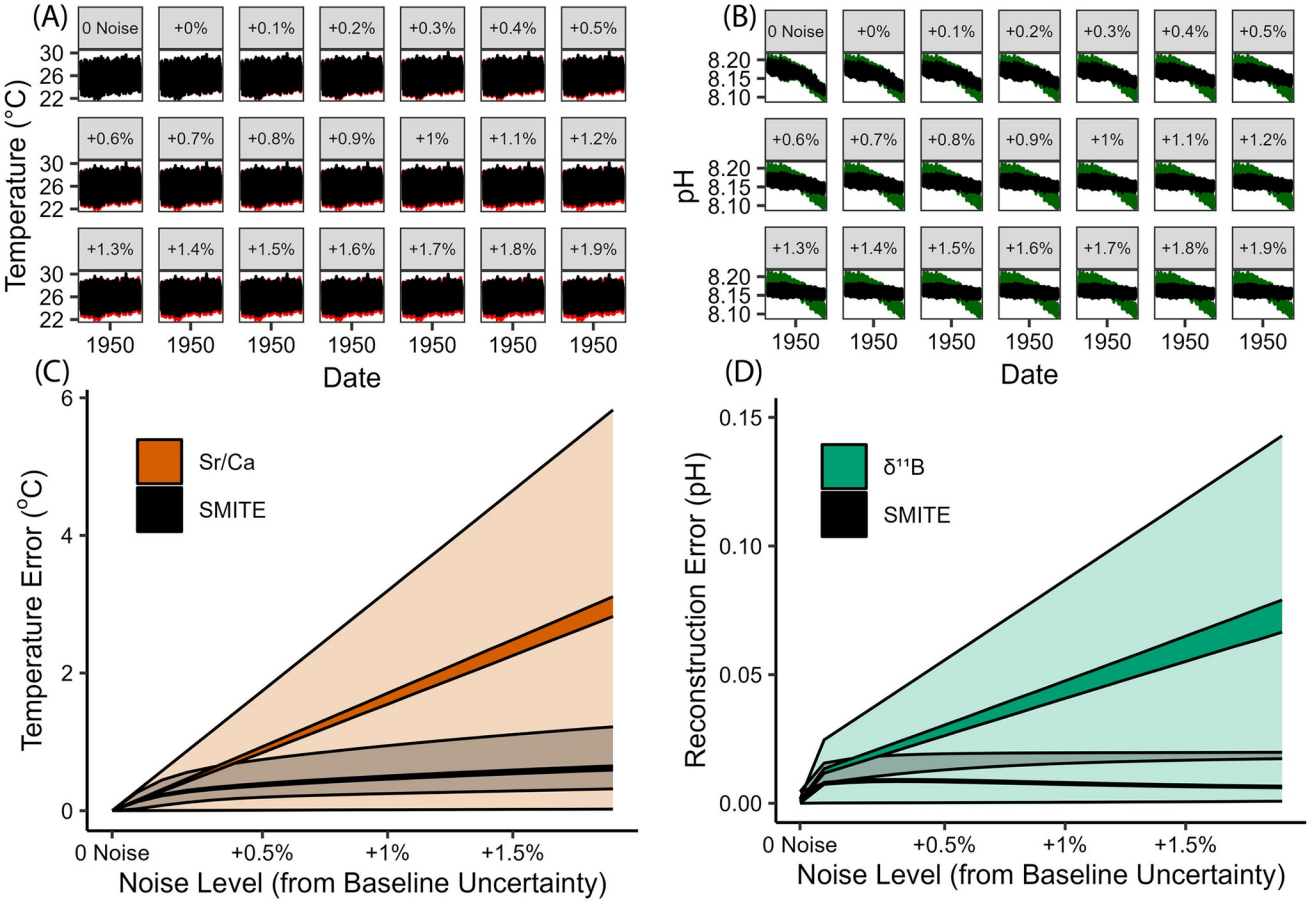
SMITE SST and pH_sw_ reconstructions for the 20th century (A-B), and reconstruction statistics for SMITE SST, Sr/Ca SST (C), SMITE pH_sw_, and *δ*^11^B pH_sw_ (D) across various levels of Gaussian noise. (A-B) Each panel represents the factor by which Gaussian (analytical) was increased in terms of relative standard deviation (RSD). Black lines represent the SMITE reconstruction for the 20th century at each noise increment. Colored lines represent the climate targets over the same period (SST = red, pH_sw_ = green). (C-D) The RMSE (translucent shaded region) and SEP (opaque shaded region) for SST and pH_sw_ estimates derived from the SMITE method are black, while the colored regions represent the RMSE and SEP for Sr/Ca SST (orange) and *δ*^11^B pH_sw_ (green). Like the panels in A and B, the x-axis represents the factor by which Gaussian (analytical) noise was increased in terms of RSD. The upper and lower bounds of each shaded region represent the maximum and minimum values for the RMSE and SEP at each noise increment.

We utilized our synthetic proxy dataset (Sr/Ca, *δ*^18^O, *δ*^11^B) to assess the relative impact of Gaussian noise on SMITE SST and pH_sw_ reconstructions (Fig 4). The top panels (A-B) show the SMITE SST and pH_sw_ reconstructions relative to their respective climate targets throughout the 20th century as we progressively increased Gaussian noise by relative standard deviation (RSD). The bottom panels (C-D) show the maximum and minimum RMSE (translucent regions) and SEP (opaque regions) for SMITE SST and pH_sw_ estimates (black), Sr/Ca SST (orange) and *δ*^11^B pH_sw_ (green) at each noise increment. We emphasize that the mean value of each synthetic proxy measurement exhibits the ideal relationship with the climate target, while the upper and lower bounds of the 95% confidence interval for each measurement (which increases as Gaussian noise increases) represent maximum deviation from the ideal. The minimum RMSEs depicted in Fig 4C and 4D (lower bounds of the translucent regions) are calculated from the idealized mean values of each measurement. In practice, these minimum RMSEs would be achieved under two conditions: a perfect relationship between the proxy and the climate target, and a near-limitless number of repeat sample analyses.

Conversely, the maximum RMSEs (the upper bounds of translucent regions) are calculated from the average RMSE between the upper and lower bounds of the 95% confidence interval for each reconstruction. They are thus representative of how high RMSEs could be, given a certain level of noise and disregarding the benefits of repeat sample analysis. Therefore, the maximum and minimum RMSEs for each reconstruction method represent two extremes. Given a certain level of noise, any study would exhibit RMSEs closer to one or extreme or the other depending on the quality of the ‘true’ relationship between the proxy and the climate target, as well as how many repeat analyses could be conducted on a particular sample. A companion assessment examining the effect of autocorrelated noise is available in the supplemental material (S1 and S2 Figs), which shows far less impact than Gaussian noise except for at very high values of the lag-1 autocorrelation coefficient (>0.9).

**Fig 5:**
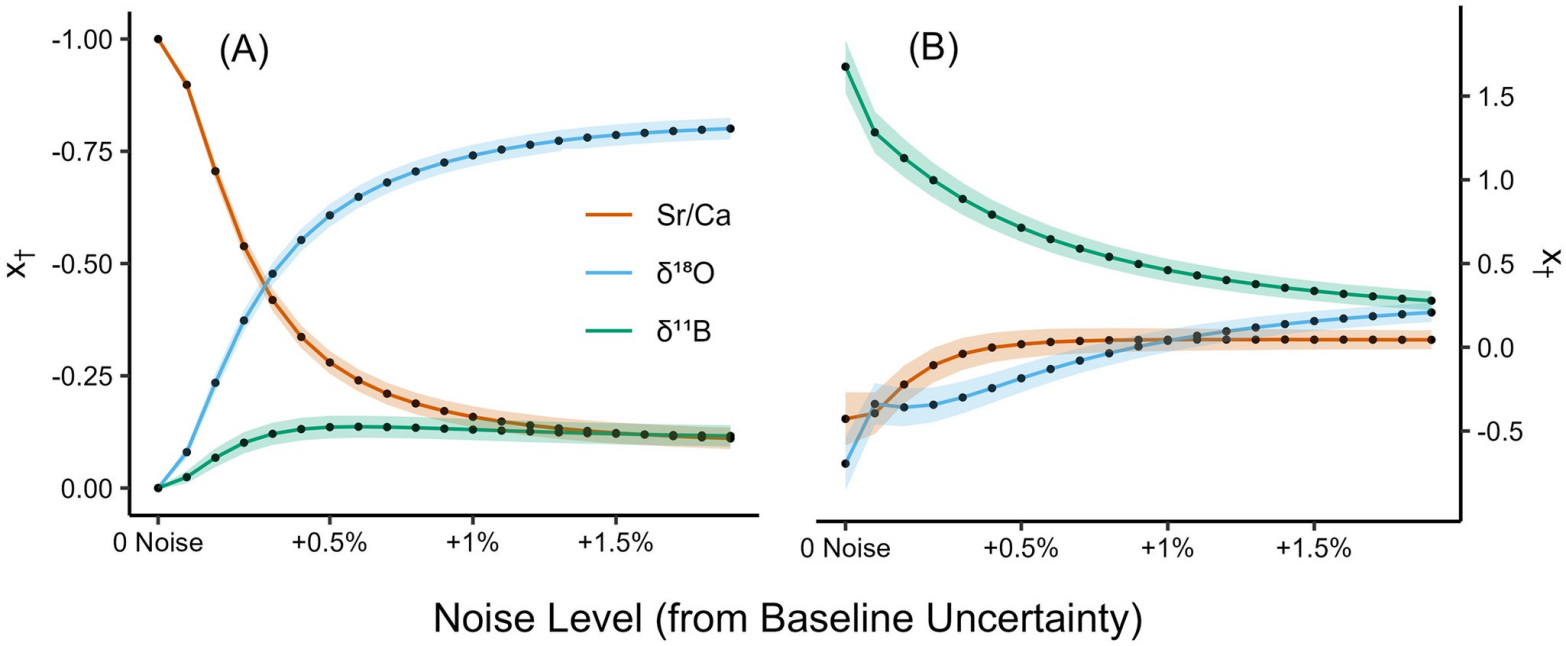
Synthetic SMITE model parameters for SST (A) and pH_sw_ (B) as Gaussian noise is increased from minimum (analytical) uncertainty in terms of relative standard deviation (RSD). The color of each line denotes the proxy associated with each model parameter (orange = Sr/Ca, blue = *δ*^18^O, green = *δ*^11^B). The shaded region around each line indicates the 95% confidence interval associated with that model parameter.

We observe from the similarities between SMITE SST estimates and ‘true’ SST in Fig 4A (red and black lines, respectively), as well as the shallow slope of the SEP and RMSE in response to noise in Fig 4C, that SMITE SST estimates in the synthetic dataset are highly robust to even exceptionally high levels of Gaussian noise. For synthetic Sr/Ca SST, the SEP and the maximum RMSE exhibit a linear relationship with Gaussian noise, which is expected given the linearity between synthetic Sr/Ca ratios and SST. At low levels of noise (< +0.2% RSD), SMITE follows this same pattern of linearity between reconstruction statistics and noise. However, as noise continues to increase, the slope of SMITE’s reconstruction statistics shoals, leading to significant improvements in SMITE SST reconstructions relative to Sr/Ca alone. This shallowing of the reconstruction statistics in response to noise can be directly attributed to SMITE’s increasing dependence on *δ*^18^O as noise increases (Fig 5), which in turn is due to the improved sensitivity of synthetic *δ*^18^O as a paleothermometer relative to Sr/Ca at higher noise levels.

For the synthetic pH_sw_ reconstructions (Fig 4B and 4D), *δ*^11^B reconstruction statistics also exhibit a linear relationship with increasing noise levels, like the reconstruction statistics observed from synthetic Sr/Ca SST. The rapid increase in pH_sw_ reconstruction error at the first noise increment (+0% RSD) can be explained by the higher baseline uncertainty for synthetic *δ*^11^B (0.40% RSD) relative to Sr/Ca (0.10% RSD), and the 0.1% RSD increase at each noise increment thereafter. We also observe that SMITE pH_sw_ SEP and maximum RMSEs remain well below that of *δ*^11^B alone, with the difference in reconstruction skill increasing as noise increases. This remains true at all noise increments, even as SMITE model parameters remain preferentially weighted towards *δ*^11^B (Fig 5B).

However, we note two key differences between the synthetic SST and pH_sw_ reconstructions. First, we observe that when no noise is implemented into the system, synthetic *δ*^11^B pH_sw_ estimates outperform those derived from SMITE by a small margin. This is due to the idealized relationship between synthetic *δ*^11^B values and pH_sw_, which follows Eqs 10 and 11. Conversely, SMITE simply treats *δ*^11^B as linearly correlated with pH_sw_ and considers its covariance with Sr/Ca and *δ*^18^O. Thus, SMITE makes no assumptions regarding the relationship between pH_sw_ and pH_cf_ nor changes *pK_B_*, which would confound the pH_sw_ signal due to interference from SST and SSS. SMITE only considers the uncertainties in the coral variable field, which we have defined at each noise increment as a percent-RSD increase from baseline uncertainty. Second, SMITE pH_sw_ estimates are far less skillful than SMITE SST estimates, particularly when considering the long-term trend as noise levels increase (Fig 4A and 4B).

The loss in both long-term and short-term variance for SMITE pH_sw_ estimates results from the compounding effects of relatively high uncertainty in synthetic *δ*^11^B, as well as the distribution of SMITE model parameters. Unlike SST (which is encoded in all three synthetic variables), information associated with pH_sw_ is only reflected in synthetic *δ*^11^B. Thus, SMITE is fully dependent on synthetic *δ*^11^B to capture the long-term trend in pH_sw_. At the first noise increment, the long-term trend in SMITE pH_sw_ estimates (∼0.085 units) is approximately 70% of the long-term pH_sw_ trend (∼0.12 units). Nearly 0.02 pH units of this 0.035-unit discrepancy can be explained by the uncertainty in synthetic *δ*^11^B (0.18‰ at the first noise increment). The remaining discrepancy of 0.015 pH units can be explained by the fact that *δ*^11^B only contributes to 57% of SMITE pH_sw_ estimates at the first noise increment (Fig 5B). The remaining 43% are divested into synthetic Sr/Ca and *δ*^18^O, both of which exhibit virtually no long-trend. Thus, only a fraction of the long-term trend in pH_sw_ captured by synthetic *δ*^11^B is propagated through the SMITE model. As noise increases throughout the experiment, the sum of the SMITE model parameters decreases, uncertainty in synthetic *δ*^11^B values continues to rise, and the resulting SMITE pH_sw_ reconstruction exhibits progressively smaller variance.

Despite the loss of long-term variance, SMITE still yields more accurate and precise predictions of pH_sw_ at all noise increments where noise is present relative to synthetic *δ*^11^B alone. This is due to the large 95% confidence interval associated with synthetic *δ*^11^B pH_sw_ estimates once noise is introduced into the system, which is propagated by the uncertainty in synthetic *δ*^11^B, SST and SSS (which influence *pK_B_* and pH_cf_), and pH_sw_. However, as mentioned before, the negative effects of the large confidence interval in synthetic *δ*^11^B can be mitigated by repeat sample analysis. Thus, given a certain number of repeat analyses according to the central limit theorem, it is possible for both synthetic *δ*^11^B pH_sw_ and synthetic Sr/Ca SST estimates to outperform estimates derived from SMITE, since minimum SMITE RMSEs remain higher than both throughout the experiment. However, in practice, this would require both an idealized relationship between the proxy and the climate target in addition to repeat sample analyses.

**Fig 6:**
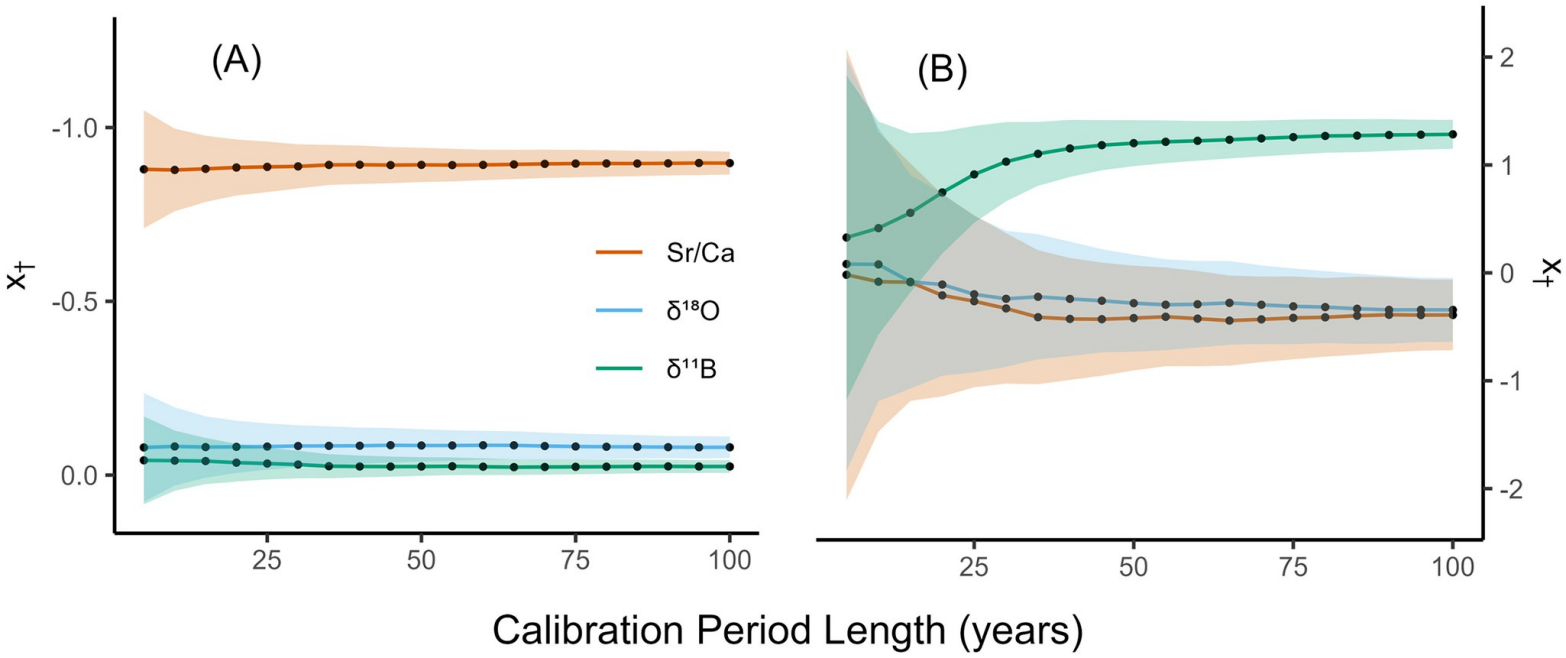
Synthetic SMITE model parameters for SST (A) and pH_sw_ (B) as the calibration period is increased from five to one hundred years in five-year increments. The color of each line denotes the proxy associated with each *x*_†_ value (orange = Sr/Ca, blue = *δ*^18^O, green = *δ*^11^B). The shaded region around each line indicates the 95% confidence interval associated with that model parameter.

We also assessed how SMITE model parameters varied with different calibration period lengths (Fig 6). In this experiment, we fixed the noise level at +0% RSD (baseline uncertainty) and increased the calibration period for the SMITE method from 5 to 100 years in five-year increments. For the SST reconstruction, *x*_†_ values for each SMITE model parameter remain fixed at -0.92 (Sr/Ca), -0.08 (*δ*^18^O), and -0.01 (*δ*^11^B) throughout the course of the experiment. This suggests that SMITE SST model parameters are stable with only five years of calibration data (the first increment we tested). Conversely, model parameters for the SMITE pH_sw_ reconstruction take approximately 30 years to stabilize due to high levels of uncertainty. Again, this is likely because *δ*^11^B is the only synthetic proxy in this dataset that is sensitive to pH_sw_, and the long-term trend in pH_sw_ increases in magnitude halfway through the 20th century.

### 3.2 Application study

**Fig 7:**
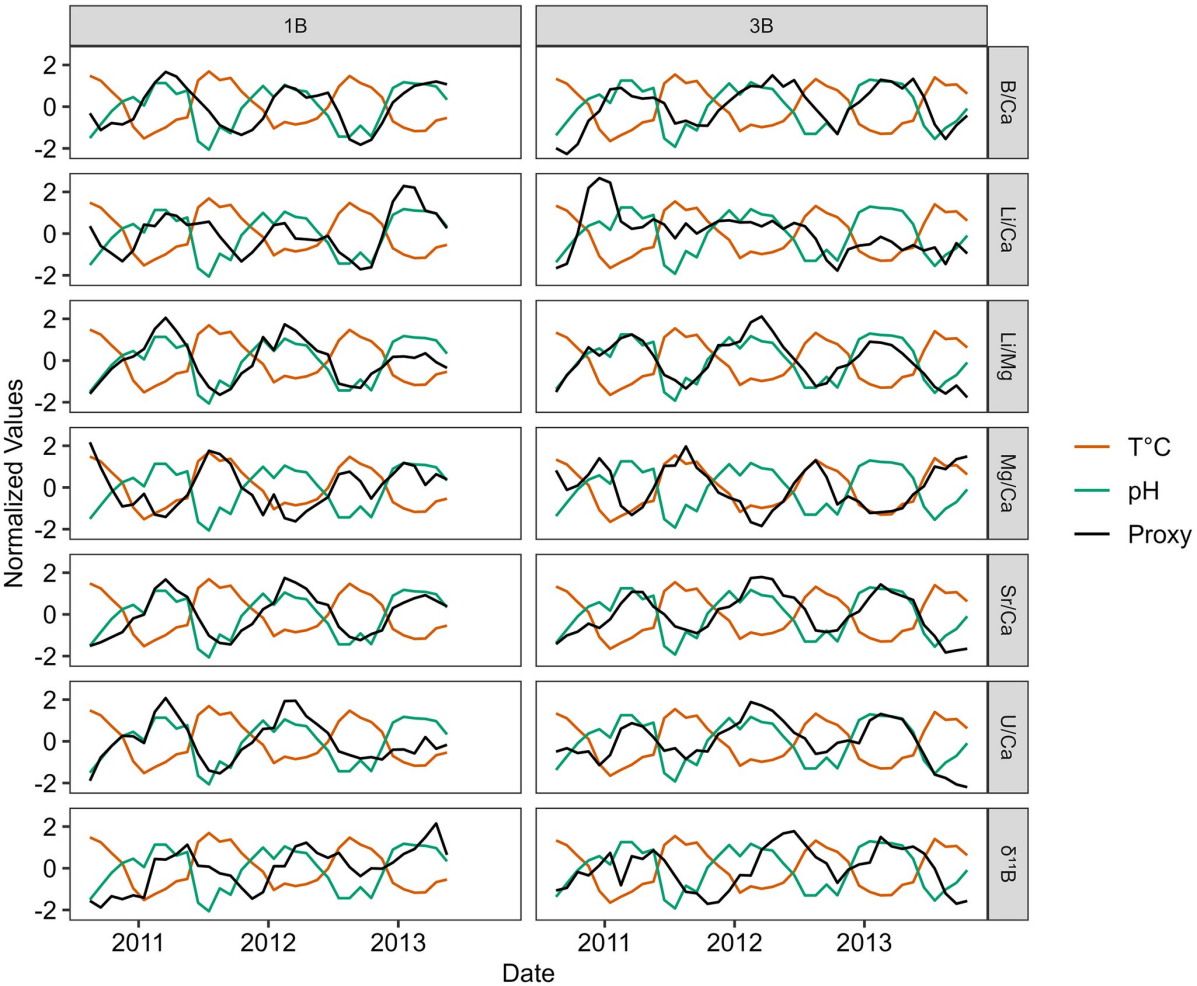
Z-score normalized monthly-averaged coral variable data (black lines) from *P. astreoides* corals 1B (left) and 3B (right). *In situ* normalized SST (orange lines) and pH_sw_ (green lines) are plotted inside each panel to show each proxy’s relative correlation to the climate target. Data from coral 1B were assigned ages between June 2010 and April 2013 (n = 35). Data from coral 3B were assigned ages between June 2010 and September 2013 (n = 40).

Seven coral skeletal geochemical variables (B/Ca, Li/Ca, Mg/Ca, Sr/Ca, U/Ca, *δ*^11^B + Li/Mg) were measured and age-modeled from both Bermudan *P. astreoides* corals between June 2010 and September 2013 (Fig 7). For ease of interpretation, each coral variable was z-score normalized (black lines) and plotted alongside mean-standardized SST (orange lines) and pH_sw_ (green line). The mean and variance of each coral variable, their respective analytical errors, and their correlations to both SST and pH_sw_ are provided in S1 Table. In both coral cores (1B and 3B), Sr/Ca and Li/Mg ratios exhibit the strongest individual correlations to SST (Sr/Ca, r = −0.85 and −0.77; Li/Mg, r = −0.83 and −0.85). B/Ca also exhibits moderately strong correlations with SST in both corals (r = −0.75 and −0.77, respectively). For pH_sw_, Sr/Ca and Li/Mg also emerge as the strongest pH_sw_ indicators, likely due to the moderate anti-correlation of SST and pH_sw_ at this site (r = −0.85).

**Fig 8:**
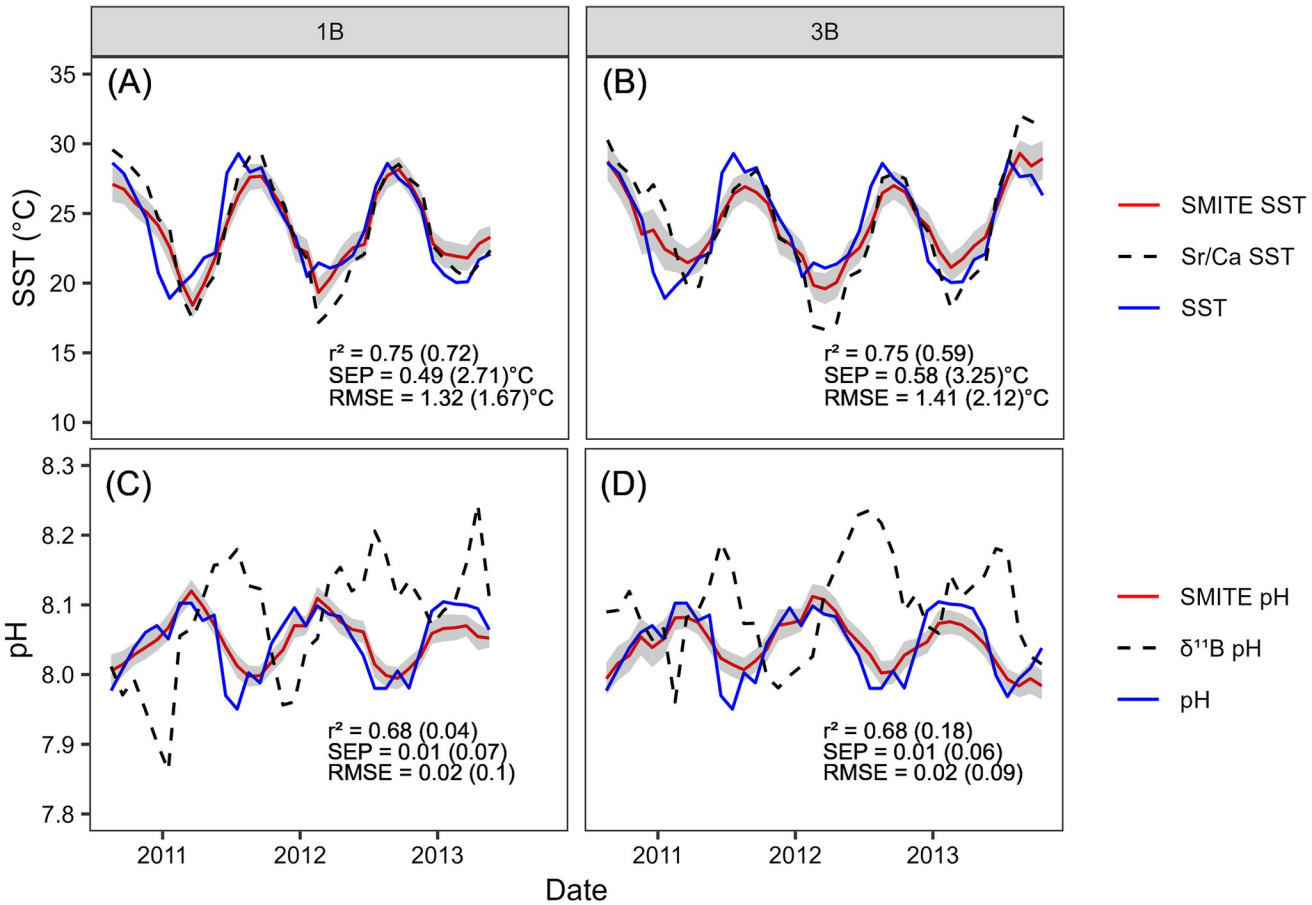
SMITE-derived SST (A-B) and pH_sw_ (C-D) estimates (red lines) from corals 1B (left) and 3B (right) compared to *in situ* SST and pH_sw_ (blue lines). SST and pH_sw_ estimates from the best or most common single-proxy estimators in these corals are plotted as dashed lines in each panel (SST = Sr/Ca; pH_sw_ = *δ*^11^B). The gray shaded region indicates the 95% confidence interval for each SMITE reconstruction. SMITE reconstruction statistics are listed at the bottom of each panel, with the equivalent statistics for Sr/Ca- and *δ*^11^B-derived estimates given in parentheticals.

SMITE SST and pH_sw_ reconstructions for corals 1B and 3B are shown in Fig 8 (red lines and shaded region). For comparison, we also plot Sr/Ca-derived SST estimates (calculated using ordinary least squares regression) and *δ*^11^B-derived pH_sw_ (calculated using Eq 10). Note that for the SMITE reconstructions, we have performed a regularization procedure that slightly reduces reconstruction skill as a tradeoff for reduced uncertainty in SMITE model parameters (Eq 7). For an in-depth discussion on the effects of this regularization procedure, see sections 3.2.2 and 4.2. For all reconstructions, the SMITE method is more accurate (RMSE), precise (SEP), and better correlated to each climate target (r^2^) than the best or most common single-proxy estimators.

Between corals 1B and 3B, SMITE SST estimates are 5 and 6 times more precise and 21% and 33% more accurate than those derived from Sr/Ca. Importantly, this notable improvement in reconstruction skill is not fully realized in the r^2^ value, where SMITE SST estimates in coral 1B are only marginally better than those derived from Sr/Ca. For the pH_sw_ reconstructions between corals 1B and 3B, the SMITE method is 7 and 6 times more precise and 5 times more accurate than *δ*^11^B. This vast improvement can largely be attributed to the relatively poor performance of *δ*^11^B as a proxy for pH_sw_ in these systems, particularly in coral 1B. However, while Li/Mg is the strongest pH_sw_ proxy in both corals, it is not often used for pH_sw_ reconstructions given its known dependence on temperature [56]. Regardless, we still observe large improvements in SMITE pH_sw_ estimates relative to Li/Mg pH_sw_ estimates, with 5 times better precision and 33% and 50% better accuracy in SMITE than from Li/Mg (S2 Table). We reiterate that this improvement is not fully realized in the correlation coefficient alone, where SMITE and Li/Mg-derived pH_sw_ estimates are comparable.

**Fig 9:**
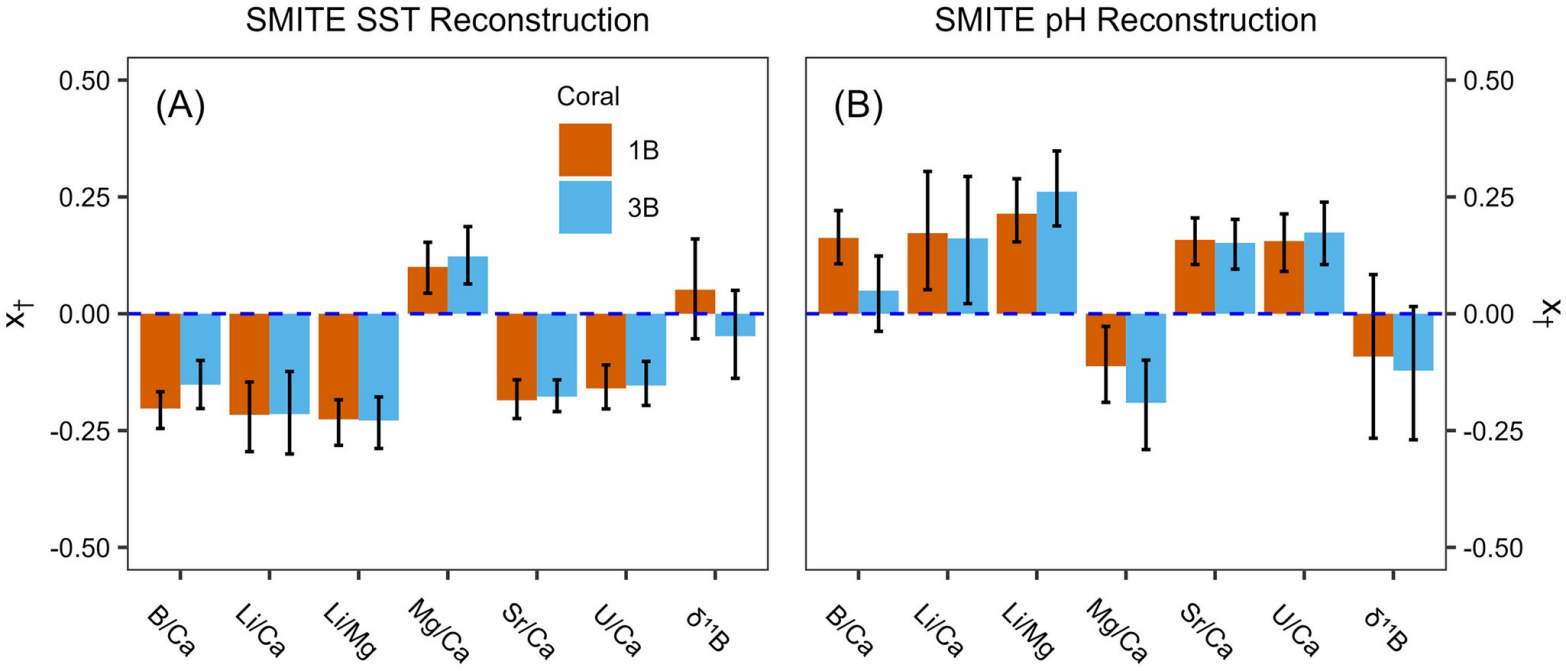
SMITE model parameters for each coral variable corresponding to the SMITE SST reconstruction (A) and pH_sw_ reconstruction (B). The loadings for each coral (1B & 3B) are indicated by the orange and blue colors, respectively. Error bars represent the 95% confidence interval for each loading.

**Fig 10:**
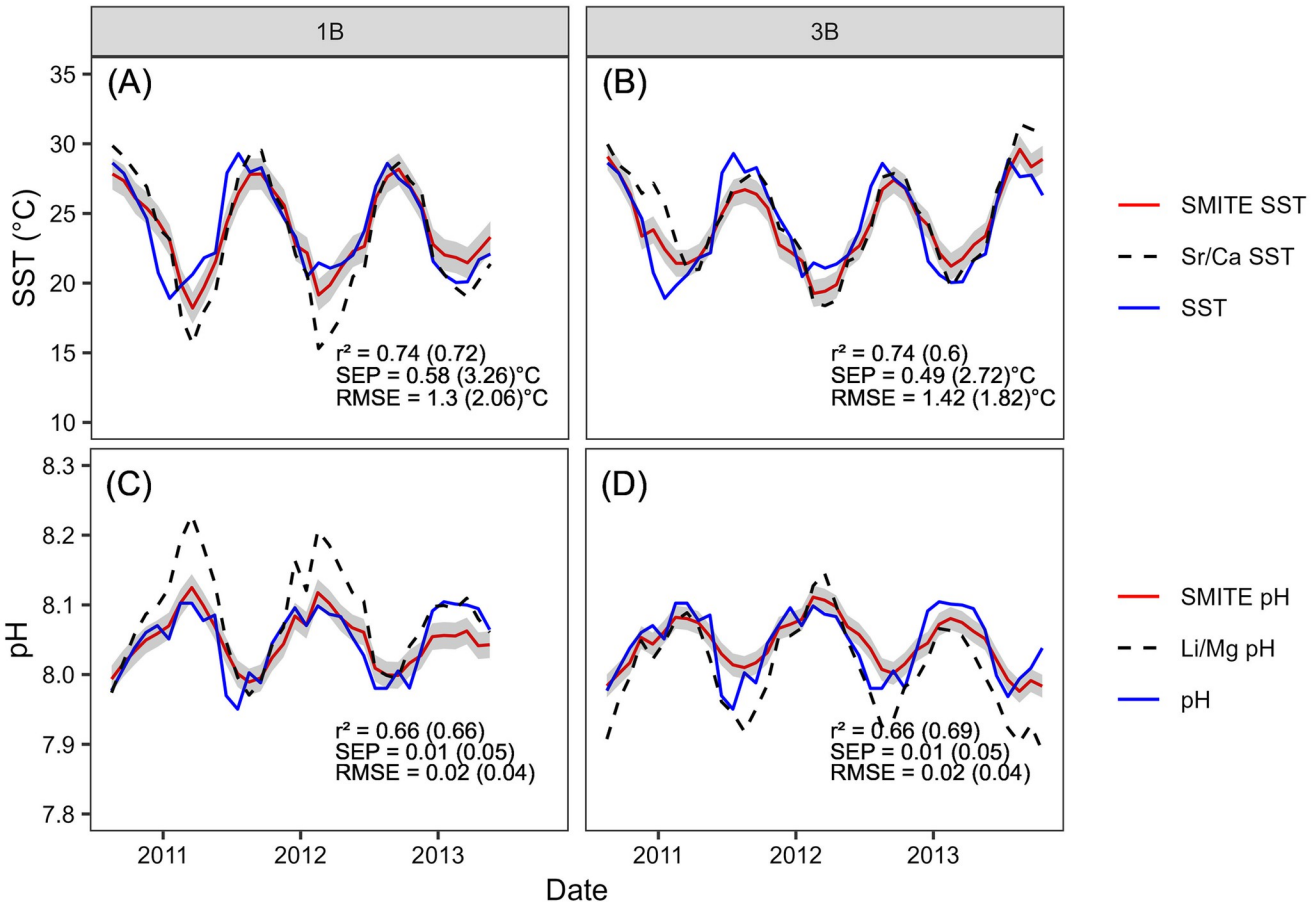
SMITE-derived SST (A-B) and pH_sw_ (C-D) estimates (red lines) compared to *in situ* SST and pH_sw_ (blue lines) when using regression coefficients derived *from the opposite corals*. SST and pH_sw_ estimates from the best single-proxy estimators in these corals are plotted as dashed lines in each panel (SST = Sr/Ca; pH_sw_ = Li/Mg). The gray shaded region indicates the 95% confidence interval for each SMITE reconstruction. SMITE reconstruction statistics are listed at the bottom of each panel, with the equivalent statistics for Sr/Ca- and Li/Mg-derived estimates given in parentheticals.

Next, we compare SMITE model parameters between corals 1B and 3B to assess their reproducibility (Fig 9). All SMITE model parameters in both the SST and pH_sw_ reconstructions are highly reproducible between corals, exhibiting *x*_†_ values that are nearly identical and/or within error of each other. Notably, the uncertainty in each SMITE model parameter tends to decrease as the absolute *x*_†_ value increases. For reproducibility, it is crucial that variables with the highest *x*_†_ values (i.e., have the strongest impact on the reconstruction) are statistically indistinguishable from one another between corals. Indeed, we see this is the case for the SMITE SST reconstruction, where *x*_†_ values associated with Li/Ca, Li/Mg, Mg/Ca, Sr/Ca, and U/Ca are nearly identical between corals. Consequently, SMITE SST estimates are highly reproducible between corals, exhibiting significantly better correlations, accuracy, and precision than the relatively more direct Sr/Ca method (Fig 10A and 10B). We also find that SMITE pH_sw_ model parameters exhibit very similar *x*_†_ values between Li/Ca, Li/Mg, Sr/Ca, and U/Ca. This results in highly skillful SMITE cross-compared pH_sw_ estimates that exhibit similar correlations with superior precision and accuracy relative to those derived from Li/Mg (Fig 10C and 10D). While this is a relatively small sample size (two corals over a few annual cycles), the level of reproducibility of both the SMITE SST and pH_sw_ reconstructions between corals is highly encouraging and warrants more rigorous testing in future studies.

### 3.3 User-based approaches for modifying SMITE reconstruction skill

#### 3.3.1 Systematic inclusion of coral variables on the SMITE SST reconstruction

**Fig 11:**
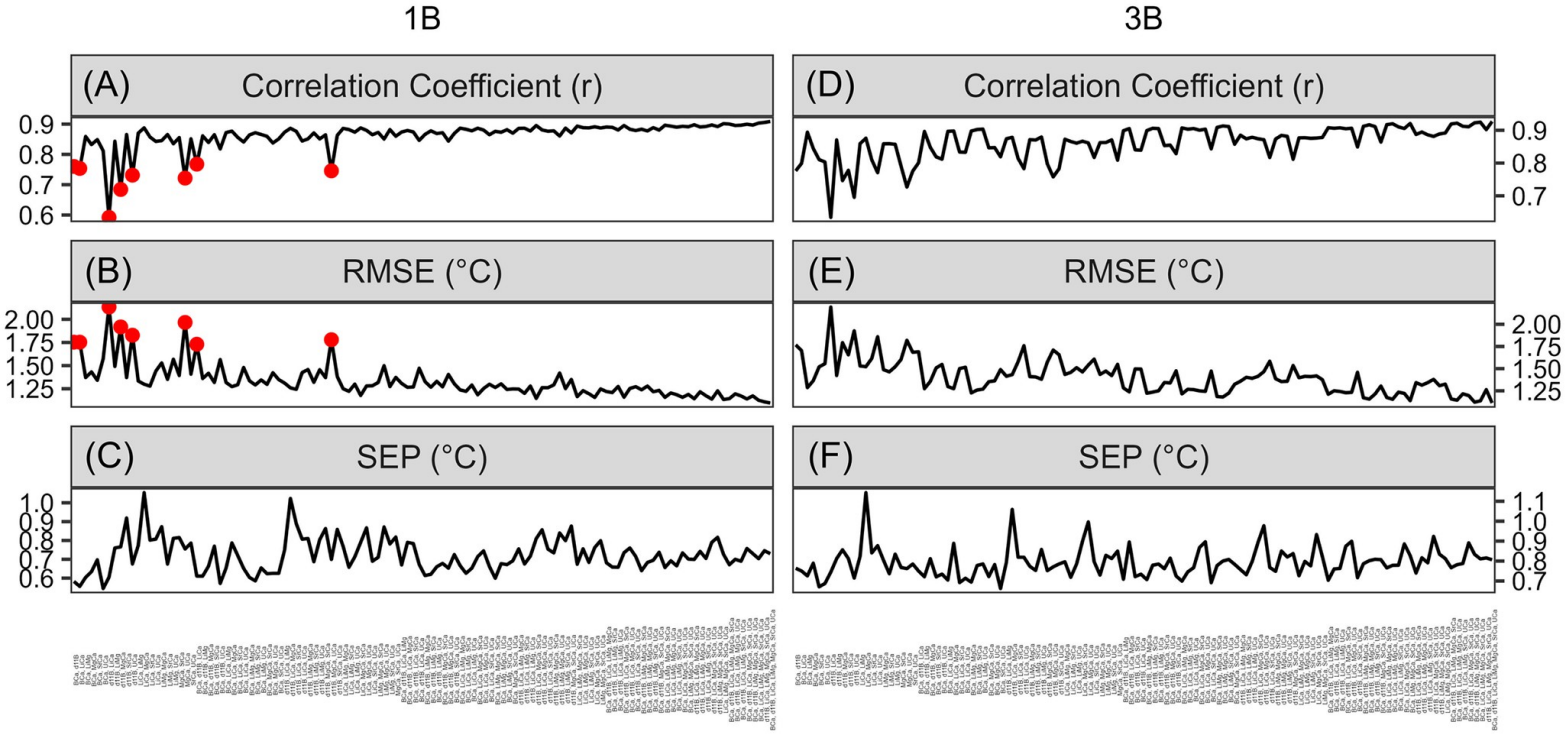
The correlation coefficient (r; A and D), the root-mean-square-error (RMSE; B and E), and standard error of prediction (SEP; C and F) for each SMITE SST reconstruction from the Bermudan *P. astreoides* corals through every combination of the seven coral geochemical variables (n = 120). The left side of each plot begins with only two coral variables (B/Ca and *δ*^11^B). Each line then tracks the corresponding reconstruction statistic as variables are systematically replaced and added to the SMITE SST reconstruction. Each line thus ends on the final value of each reconstruction statistic when all seven coral variables are used.

An intuitive procedure that new SMITE users might implement to optimize reconstruction skill would be to selectively exclude certain coral variables. Therefore, we applied the SMITE method to every possible combination of 2–7 coral variables (n = 120) in the two Bermudan corals and examined the subsequent impact on the correlation (r), accuracy (RMSE), and precision (SEP) of the SMITE SST reconstruction (Fig 11). This experiment was performed with no regularization implemented (Eq 7). We also provide a complimentary analysis regarding SMITE pH_sw_ reconstructions in the supplemental information (S3 Fig).

Throughout the experiment, two notable patterns emerge. First, we observe a small but statistically significant trend (*p* < 1*e*−9) towards higher r-values and lower RMSEs for both corals as more variables are added to the SMITE method. Second, we note that there are certain combinations of coral variables that yield poorer quality reconstructions, indicated by ‘spikes’ in each of the reconstruction statistics. These spikes are particularly noticeable in coral 1B, which interestingly coincide with when both Li/Mg and Sr/Ca are excluded from the model (Panels A and B, red points). Furthermore, we note that the magnitude of these spikes decreases significantly as more coral variables are added to the SMITE SST reconstruction.

#### 3.3.2 Regularization through truncation of singular values

**Fig 12:**
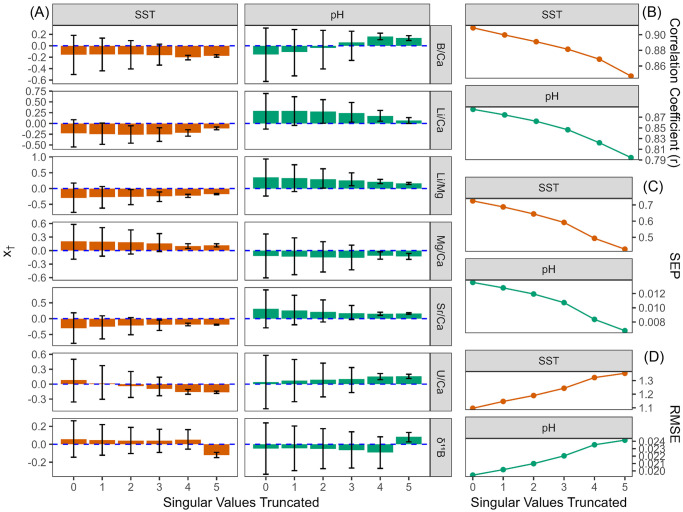
The effects of truncating singular values from coral 1B on the SMITE SST and pH_sw_ reconstruction. The x-axis in each plot denotes the number of singular values truncated. Each plot thus shows the progressive effects from no truncation (left) to maximum truncation (right). Truncation occurs from the highest (least dominant) singular values to the lowest (most dominant) singular values. The first two singular values can never be truncated. Colors distinguish the results from the SST reconstructions (orange) versus the pH_sw_ reconstructions (green). (A) SMITE model parameters, or *x*_†_ values, at each successive level of truncation. Rows denote the SMITE model parameter. The colored bar within each plot indicates the *x*_†_ value of the corresponding SMITE model parameter at a given level of truncation. Error bars for each *x*_†_ value denote the 95% confidence interval based on a Monte Carlo approach. (B—D) The correlation coefficient (r; B), the standard error of prediction (SEP; C), and the root-mean-square-error (RMSE; D) at each successive level of truncation.

SMITE method users can reduce uncertainty in SMITE model parameters by removing, or ‘truncating’, higher order (or less dominant) singular values from the coral variable field dataset (Eq 7). Here we demonstrate the effects of sequentially truncating singular values on the coral 1B SMITE SST and pH_sw_ reconstructions (Fig 12). The singular values of both Bermudan coral datasets are shown in S4 Fig, and a complimentary regularization analysis of coral 3B is provided in S5 Fig. At first glance, regularization has similar effects between the SMITE SST and pH_sw_ reconstructions. With each singular value truncated, uncertainty with respect to each SMITE model parameter is reduced, the correlation coefficient and SEP decrease, and the RMSE increases. In short, regularization compromises accuracy and correlation in exchange for precision and reduced uncertainty in SMITE model parameters. The optimum level of regularization depends on two factors. First, the size of the coral dataset determines how many singular values can be truncated. Thus, a dataset with a greater number of variables offers greater control over how much information users could omit from the calibration. Second, regularization may vary depending on how information related to the climate target of interest is spread among the singular values.

It is important to note that results from the synthetic dataset are shown with no regularization implemented (section 1). With only three coral variables in the dataset (Sr/Ca, *δ*^18^O, *δ*^11^B) and three potential climate targets (SST, SSS, pH_sw_), only one singular value can be truncated from the synthetic dataset. As expected, truncating the highest order singular value in the synthetic dataset results in greatly reduced uncertainty in SMITE model parameters that, in the case of SST, are more stable across noise treatments. However, we also observe a significant reduction in reconstruction skill. This is likely because in a square system such as this (i.e., an equal number of climate targets and predictor variables), important climate information could be stored in even the highest order singular value. Therefore, we recommend only implementing truncation in overdetermined systems, where the number of coral variables is significantly higher than the number of climate targets.

## 4 Discussion

Here we used a variety of data types to assess the quality of SST and pH_sw_ estimates derived from the SMITE method, a novel multivariate calibration method that leverages covariance across a coral variable field to optimize reconstruction skill. Using two Bermudan *P. astreoides* corals as a proof-of-concept, we show that SMITE SST and pH_sw_ estimates are more accurate and precise than those derived from the best single-proxy estimators in each system. Furthermore, we find a high degree of inter-colony reproducibility in SMITE model parameters, and consequently reconstruction skill, when the SMITE method is applied to two corals that were collected near one another under identical environmental conditions. The reproducibility of SMITE model parameters across cores, which is achieved at higher levels of regularization (i.e., more singular values truncated), suggests that this method may hold great promise for application to the fossil record (i.e., when no calibration data are available for a particular core). In this section, we will discuss why the inclusion of different coral variables and regularization exhibit their observed effects on the accuracy, precision, correlation, and reproducibility of each reconstruction in the Bermuda coral datasets. As we examine the effects of these user-controlled procedures, we will use the results from the synthetic proxy studies to better inform our discussion of their effects on reconstruction skill. We conclude this section with a list of recommendations for those who wish to implement the SMITE method in their own datasets.

### 4.1 Properties of the coral variable field and their effects on SMITE reconstruction skill

Our findings in this study suggest that the quality of the SMITE reconstruction is contingent on both the quality and quantity of coral variables included in the reconstruction. The quantity effect is most clearly observed in the systematic inclusion experiment, where SMITE reconstruction skill improves as more coral variables are added to the SMITE dataset. However, there is a sense of ‘diminishing returns’ with how much the reconstruction improves after a certain threshold. The exact position of this ‘threshold’ warrants more rigorous testing in future studies. We observe the effect of coral variable quality in this same systematic inclusion experiment, specifically in the ‘baseline’ stability of the SMITE reconstruction (i.e., the overall variance of the reconstruction statistics throughout the experiment). We hypothesize that this baseline stability is a function of the coral variables with the strongest relationship to the climate target. For coral 1B, the presence of two robust SST proxies (Sr/Ca and Li/Mg) leads to many different combinations of variables that produce strong SMITE SST reconstructions. Therefore, the main source of variance in the correlation and accuracy of the reconstructions is observed when neither Sr/Ca nor Li/Mg are included in the reconstruction (red dots, Fig 11). While SMITE SST reconstructions for coral 3B still exhibit a high degree of skill throughout the course of the experiment, the greater variability we observe in the baseline SST reconstruction may be due to the relatively poorer performance of Sr/Ca, since Li/Mg remains a strong SST proxy in both corals.

We see further evidence for the importance of both the quantity and quality of the coral variables in the SMITE dataset when we consider how Gaussian noise impacts the synthetic proxy dataset. At a noise factor of 0, we observed how Sr/Ca and *δ*^11^B were the greatest contributors to the SMITE SST and pH_sw_ reconstructions, respectively (Fig 5). This is logical, as synthetic Sr/Ca and *δ*^11^B exhibit the strongest correlations with SST and pH_sw_, respectively. As noise increased, the SMITE SST reconstructions maintained a high level of skill due to the additional presence of *δ*^18^O as a very strong and sensitive SST proxy. However, SMITE pH_sw_ estimates did not capture the long-term trend after only a relatively small increase in noise. This stark contrast in reconstruction skill is due to the presence of multiple strong SST proxies in the synthetic dataset (Sr/Ca and *δ*^18^O), with the added benefit of *δ*^18^O being very robust to analytical noise. Conversely, synthetic *δ*^11^B exhibits only a moderate correlation with pH_sw_ (r^2^ = 0.68). Because SMITE treats *δ*^11^B as linearly correlated to pH_sw_ and does not consider the relationship between pH_sw_ and pH_cf_ nor changes in *pK_B_*, SMITE pH_sw_ reconstructions were significantly less skillful than the SST reconstructions. Therefore, even though we used the same exact calibration scheme for both climate target reconstructions, the stronger and more numerous SST proxies in the synthetic dataset result in stronger SMITE SST reconstructions relative to pH_sw_.

### 4.2 The effects of truncation on SMITE reconstruction skill

SMITE reconstruction skill is also dependent on the degree of regularization, or the number of singular values truncated from the coral variable field (Eq 7; Fig 12). The underlying principle behind the SMITE method is that information associated with the climate target is encoded as covariance between the coral variables. Thus, climate target information is spread out between the singular values of the coral variable dataset. For coral 1B, we find multiple lines of evidence suggesting that most of the SST information is stored in the lower order, or dominant, singular values (i.e., singular values in which SST describes a large portion of variance in the data). First, the *x*_†_ value for most SMITE model parameters remains relatively constant at all levels of regularization, with U/Ca and *δ*^11^B as the exception. Second, accuracy and correlation decrease linearly from 0–4 singular values truncated, after which the values continue to decrease less linearly. We also note a significant reduction in SMITE model parameter uncertainty at this same level of truncation. These observations suggest that removing the upper four singular values (i.e., truncation level 4) removes a significant amount of non-SST-related variability from the system, thus preventing SMITE from overfitting model parameters to potential noise. We find further evidence for this interpretation when examining the singular values of the coral 1B dataset (S4 Fig), where we observe a subtle drop-off between the third singular value (74% cumulative variance explained) and the fourth singular value (82% cumulative variance explained). This drop-off also exists in the coral 3B dataset, with only minor differences in cumulative variance explained (69% and 80%, respectively). All these lines of evidence suggest that, for both the 1B and 3B SMITE SST reconstructions, the optimum trade-off of reconstruction skill and model parameter uncertainty occurs once the higher four singular values are truncated. Thus, this was the chosen level of regularization for both the 1B and 3B SMITE SST reconstructions in section 2.

For the SMITE pH_sw_ reconstruction, we find that pH_sw_ information is also likely stored in the lower order singular values. However, we note that the only SMITE pH_sw_ model parameters that do not change significantly throughout the experiment are Li/Mg, Mg/Ca, and Sr/Ca. These model parameters also have the least amount of uncertainty associated with them at each level of truncation. We also observe that the same shift in model parameter uncertainty and reconstruction skill that occurs in the SMITE SST reconstructions occurs in the SMITE pH_sw_ reconstructions at the exact same level of truncation (4). This finding further supports that the higher four singular values contain non-climate-related variability, specifically pH_sw_ in this case. Thus, we truncated the higher four singular values for the SMITE pH_sw_ reconstructions in section 2 as well. However, we speculate that the higher degree of uncertainty in SMITE pH_sw_ model parameters indicates that covariance in this particular coral variable field is not as strongly influenced by pH_sw_ as it is by SST.

### 4.3 Recommendations for implementers of the SMITE method

The results presented in this study show that SMITE SST and pH_sw_ estimates are more accurate, precise, and better correlated to *in situ* SST and pH_sw_ than those derived from the best or most-commonly-used single- and dual-proxy estimators. Furthermore, SMITE model parameters are highly reproducible between both Bermudan corals, and synthetic SMITE SST model parameters are stable across a large range of calibration periods (in this study, 5 to 100 years). The stability and reproducibility of the SMITE method makes it a promising candidate for fossil coral applications, where model parameters derived from modern cores would be applied to fossil material. Moreover, the SMITE method is computationally inexpensive and can be readily implemented into any paleoclimate reconstruction where multivariate coral data is available. Therefore, we provide here a list of recommendations for those who wish to utilize the SMITE method in their own datasets. Within this list, we provide some promising directions and potential limitations of the SMITE method for future studies.

**Include as many coral variables as possible.** While it may seem counterintuitive to include coral variables that are weakly associated with the climate target, our results show that SMITE reconstruction skill increases as the number of predictor variables increases. Additionally, increasing the number of coral variables pushes the system towards overdetermination, which increases flexibility when it comes to choosing the appropriate level of regularization. However, our results also indicate that SMITE reconstruction skill is contingent upon the performance of the best predictors of the climate target. We therefore recommend users carefully examine the relationship between each coral variable and the climate target(s) prior to implementing the SMITE method (e.g., via scatter plots, covariance structure, etc.). Coral variables may need to be transformed for optimum results, as the SMITE method is a linear regression and thus assumes linearity between the predictor variables and the climate target. Therefore, certain coral variables that exhibit a non-linear relationship to the climate target (e.g., Li/Mg to SST over wide temperature ranges) may need to be transformed prior to entry in the calibration dataset.

**Optimum regularization.** Given the high level of uncertainty in SMITE model parameters when no singular values are truncated, we recommend users implement some level of regularization when applying the SMITE method in an overdetermined system (i.e., more coral variables than climate targets). Our results show that, even at high levels of regularization in such an overdetermined system, there is a minimal trade-off in reconstruction skill for large improvements in SMITE model parameter uncertainty, which in turn results in better reproducibility between corals. Future studies could apply the SMITE method to fossil coral material and potentially improve upon existing reconstructions. To determine the optimum level of regularization, users should qualitatively assess the distribution of the singular values in the coral dataset. Users should also systematically assess the reconstruction skill and model parameter uncertainty at each level of truncation (e.g., Fig 12). We recommend implementing regularization at the point when (a) the uncertainty associated with SMITE model parameters decreases significantly; and (b) when there is an inflection point in the distribution of the singular values (for the Bermuda corals, this occurred at ∼70% cumulative variance explained).

**The degree of improvement in SMITE reconstruction skill over conventional calibration methods depends on the quality of the calibration dataset.** Without regularization, it is mathematically impossible for the SMITE method to perform worse than the single best linear predictor of the climate target in any coral variable dataset. This is because as the performance of the best predictor in the dataset improves, SMITE utilizes that information and improves accordingly. However, our results indicate that, to a point, the magnitude of the improvement in SMITE reconstruction skill over conventional univariate regression techniques increases as the quality of the coral dataset decreases. If the best predictor for the climate target in the coral variable dataset is very robust (e.g., Sr/Ca to SST in coral 1B), then SMITE’s improvement on that reconstruction will be modest. In contrast, SMITE drastically improves the reconstruction for datasets where the best predictor variable is relatively weak (e.g., *δ*^11^B to pH_sw_ in the synthetic dataset), or where the signal-to-noise ratio is relatively low (e.g., Sr/Ca to SST in the synthetic dataset when analytical noise is increased). This is extremely promising for potential SMITE SST reconstructions implemented on tropical corals utilizing ICP-MS methods, where the annual SST range is relatively small (∼2–3°C) and where the analytical uncertainty for Sr/Ca is many factors higher than for ICP-OES. However, we acknowledge that the impact of noise on a tropical coral variable dataset may not scale linearly (as modeled in the synthetic experiment) due to increased age-model uncertainties when the annual SST range is subdued. These nonlinear processes could influence the robustness of SMITE reconstructions. Thus future work is needed to address the utility of SMITE SST reconstructions using tropical corals. Additionally, for climate targets such as pH_sw_ where the linear correlation to a coral variable is relatively poor and covariance associated with the climate target may not be as pronounced, we speculate that users will need to be more strategic in which coral variables are included in the SMITE calibration dataset. Which specific coral variables are included for SMITE pH_sw_ reconstructions in particular should be rigorously tested in future studies.

**Further testing is needed for the SMITE method’s ability to capture long-term trends in pH_sw_.** Using the synthetic dataset, we show that SMITE SST model parameters are insensitive to the length of the calibration period between 5 and 100 years (in five-year increments). Conversely, SMITE pH_sw_ loadings did not stabilize until 30 years of calibration data were available. This is an unrealistic calibration period length for most coral-based pH_sw_ reconstruction. Unfortunately, SMITE pH_sw_ reconstructions calculated prior to the 30-year calibration period fail to capture the long-term trend in pH_sw_. This could be due to the complications associated with SMITE treating *δ*^11^B as a linear proxy for pH_sw_ and not accounting for variability in *pK_B_*, which is both temperature and salinity dependent. Alternatively, it could be due to the rapid decrease in pH_sw_ partway through the 20th century. While it is encouraging that SMITE pH_sw_ estimates were highly skillful and reproducible between the Bermuda corals, this could be due to either the strong covariance of pH_sw_ and SST at Hog Reef (r^2^ = 0.82), or the relatively small sample size of the Bermuda coral dataset. Thus, it cannot be ruled out that significant changes in long-term trends may further complicate the SMITE pH_sw_ reconstruction, at least in square systems when little-to-no regularization can be implemented. Furthermore, the *P. astreoides* coral datasets in this study were relatively short (35 and 40 months, respectively), and the synthetic dataset is highly idealized in its relationships between the synthetic proxies and the reconstruction targets. Additional testing of the SMITE method is needed on multi-decadal coral datasets at lower sampling resolutions (e.g., annual rather than monthly), which could elucidate how SMITE can help with broader climate interpretations.

**SMITER—an open-source package in R for implementing the SMITE method on coral datasets.** The SMITE method is computationally inexpensive and can be readily performed on a variety of statistical software programs. However, we aim to facilitate rapid dissemination of the SMITE method with the release of the open-source SMITER package in R (https://hphughescraft.github.io/SMITER). The SMITER package gives researchers a user-friendly tool to implement the SMITE method in their own datasets, which could quickly advance the community’s understanding of SMITE’s utility on tropical corals and its ability to capture long-term trends in pH_sw_, as well as how the method performs on longer datasets with coarser sampling resolution. Furthermore, application of the SMITE method on the large (and growing) number of existing coral records will generate an equally large number of SMITE model parameters. These model parameters can be independently assessed to determine the extent of the SMITE method’s reproducibility among corals of varying taxa and location. Such reproducibility-focused research questions could ultimately improve coral-based paleoclimate reconstructions on fossil material.

## Supporting information

S1 Fig Reconstruction statistics for SMITE SST, Sr/Ca SST (A), SMITE pH_sw_, and *δ*^11^B pH_sw_ (B) across various levels of autocorrelated noise. The RMSE (translucent shaded region) and SEP (opaque shaded region) for SST and pH_sw_ estimates derived from the SMITE method are black, while the colored regions represent the RMSE and SEP for Sr/Ca SST (orange) and *δ*^11^B pH_sw_ (green). The x-axis represents the factor by which autocorrelated noise was increased in terms of RSD. The upper and lower bounds of each shaded region represent the maximum and minimum values for the RMSE and SEP at each noise increment.
(TIF)

S2 Fig Synthetic SMITE model parameters for SST (A) and pHsw (B) as the lag-1 autocorrelation coefficient of the noise term is increased. The color of each line denotes the proxy associated with each model parameter (orange = Sr/Ca, blue = *δ*^18^O, green = *δ*^11^B). The shaded region around each line indicates the 95% confidence interval associated with that model parameter.
(TIF)

S3 Fig The correlation coefficient (r; A and D), the root-mean-square-error (RMSE; B and E), and standard error of prediction (SEP; C and F) for each SMITE pH_sw_ reconstruction from the Bermudan *P. astreoides* corals through every combination of the seven coral geochemical variables (n = 120). The left side of each plot begins with only two coral variables (B/Ca and *δ*^11^B). Each line then tracks the corresponding reconstruction statistic as variables are systematically replaced and added to the SMITE pH_sw_ reconstruction. Each line thus ends on the final value of each reconstruction statistic when all seven coral variables are used.
(TIF)

S4 Fig The singular values from the two Bermudan *P. astreoides* corals. The red error bars around each point indicate the 95% confidence interval estimated using a bootstrap Monte Carlo approach.
(TIF)

S5 Fig The effects of truncating singular values from coral 3B on the SMITE SST reconstruction. The x-axis in each plot denotes the number of singular values truncated. Each plot thus shows the progressive effects from no truncation (left) to maximum truncation (right). Truncation occurs from the highest (least dominant) singular values to the lowest (most dominant) singular values. The first two singular values can never be truncated. Colors distinguish the results from the SST reconstructions (orange) versus the pH_sw_ reconstructions (green). (A) SMITE model parameters, or *x*_†_ values, at each successive level of truncation. Rows denote the SMITE model parameter. The colored bar within each plot indicates the *x*_†_ value of the corresponding SMITE model parameter at a given level of truncation. Error bars for each *x*_†_ value denote the 95% confidence interval based on a Monte Carlo approach. (B—D) The correlation coefficient (r; B), the standard error of prediction (SEP; C), and the root-mean-square-error (RMSE; D) at each successive level of truncation.
(TIF)

S1 Table Mean (*μ*), standard deviation (*σ*), analytical error (*ϵ*), and correlation coefficient (r) to SST and pH_sw_ for each coral variable measured in both Bermudan *P. astreoides* corals (1B and 3B). (DOCX)

S2 Table Reconstruction statistics for pH_sw_ reconstructions in both Bermudan *P. astreoides* corals (1B and 3B). (DOCX)
