# Supplementary figures and images for: Synthetic and practical reconstructions of SST and seawater pH using the novel multiproxy SMITE method
Source: PLoS One. 2024 Jun 25;19(6):e0305607. doi: 10.1371/journal.pone.0305607 (PMC11198822; doi:10.1371/journal.pone.0305607)

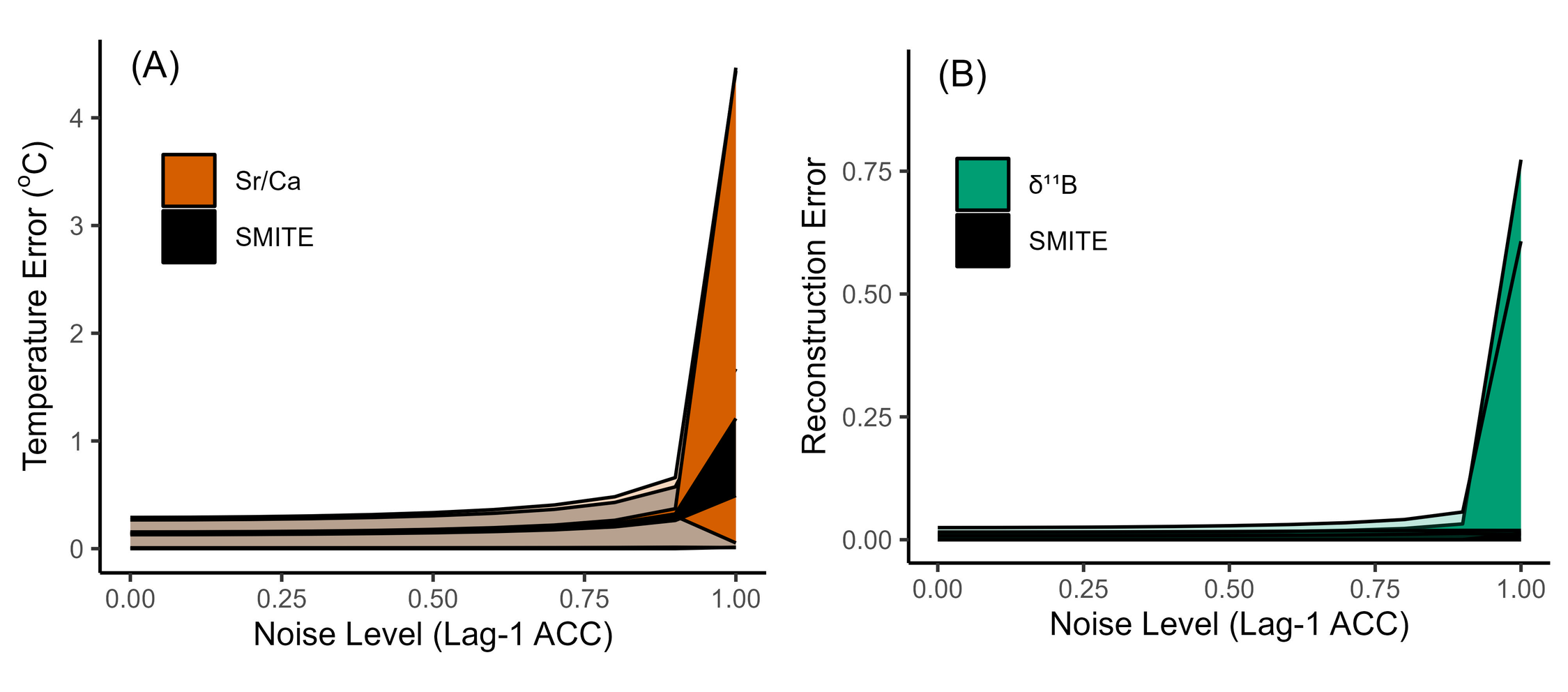

Supplement: S1 Fig — The RMSE (translucent shaded region) and SEP (opaque shaded region) for SST and pHsw estimates derived from the SMITE method are black, while the colored regions represent the RMSE and SEP for Sr/Ca SST (orange) and δ11B pHsw (green). The x-axis represents the factor by which autocorrelated noise was increased in terms of RSD. The upper and lower bounds of each shaded region represent the maximum and minimum values for the RMSE and SEP at each noise increment. (TIF) [file pone.0305607.s001.tif]

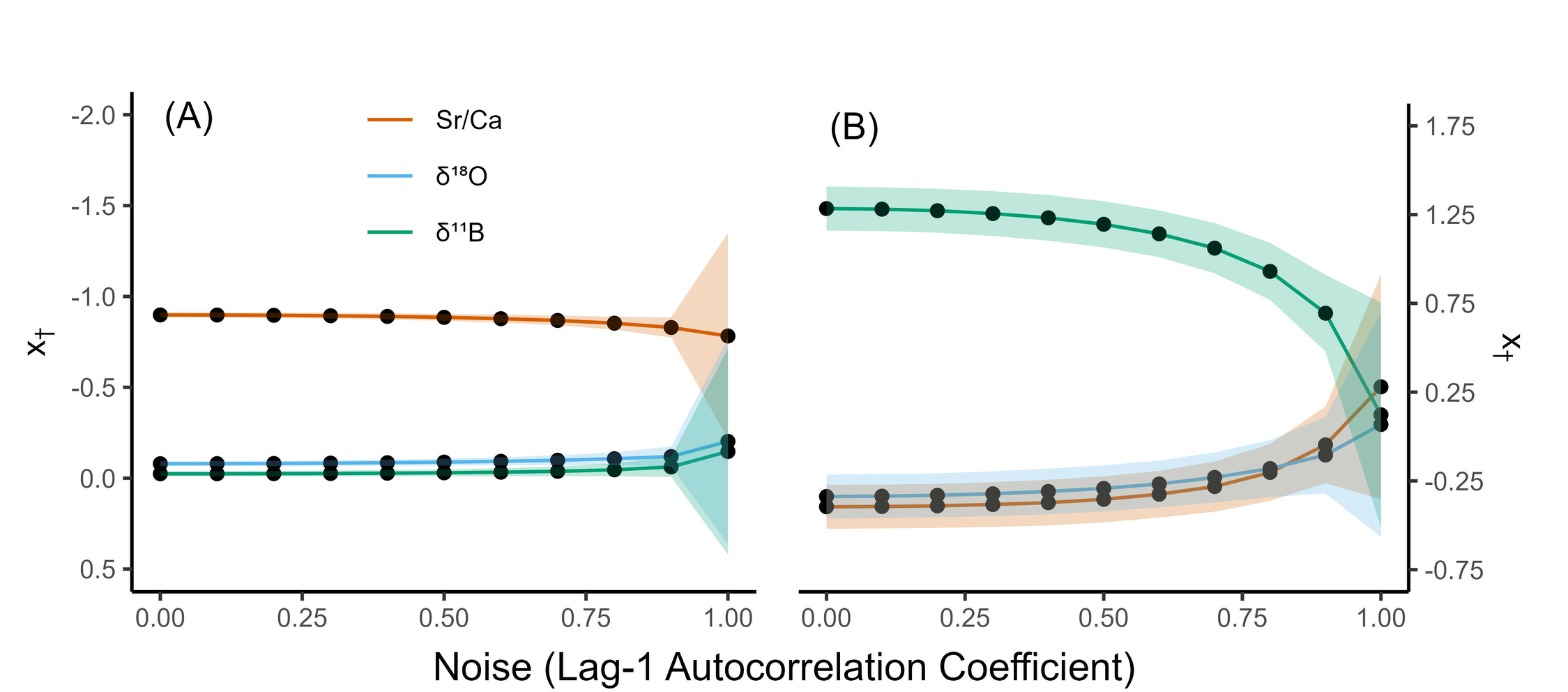

Supplement: S2 Fig — The color of each line denotes the proxy associated with each model parameter (orange = Sr/Ca, blue = δ18O, green = δ11B). The shaded region around each line indicates the 95% confidence interval associated with that model parameter. (TIF) [file pone.0305607.s002.tif]

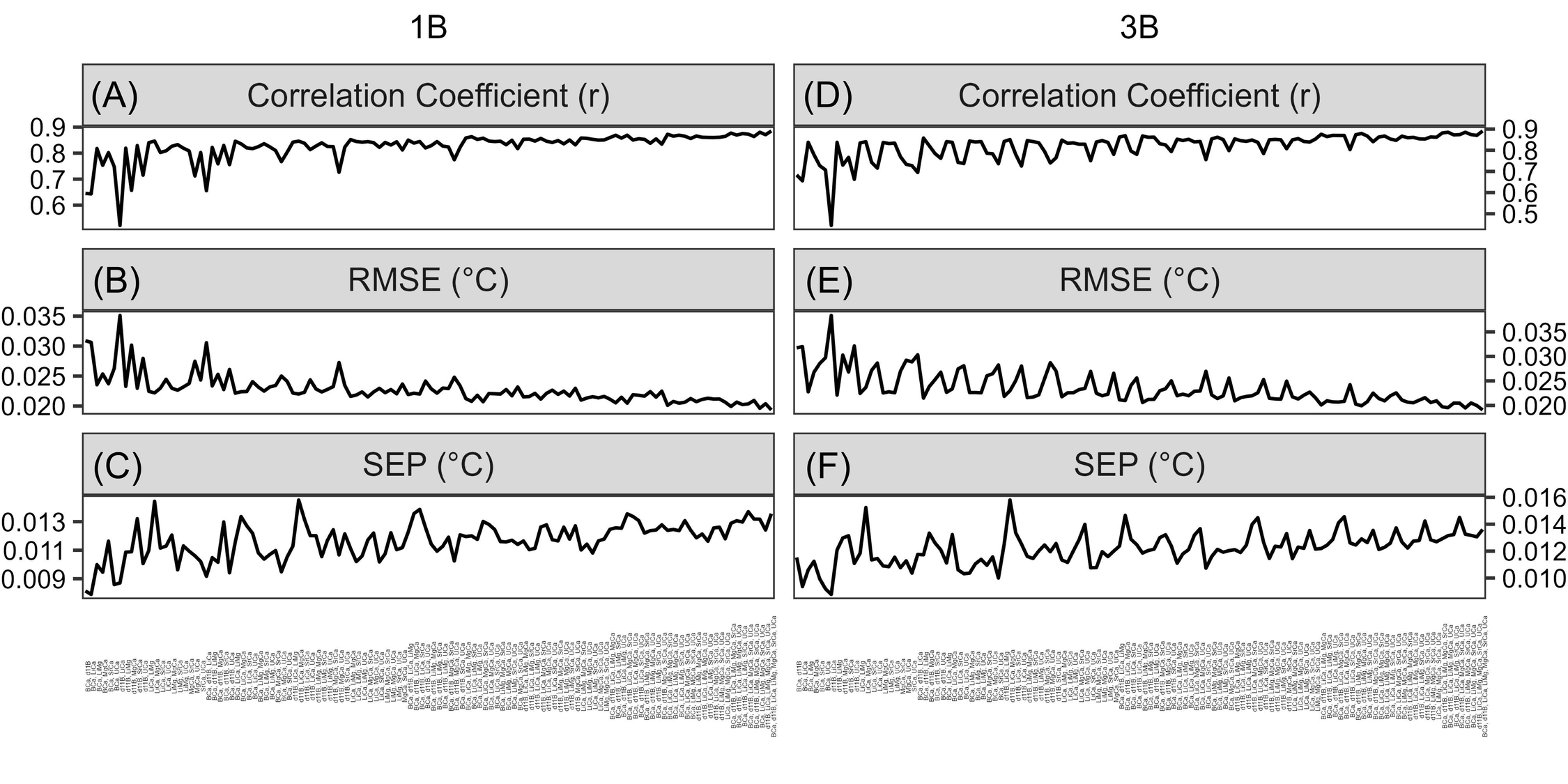

Supplement: S3 Fig — The left side of each plot begins with only two coral variables (B/Ca and δ11B). Each line then tracks the corresponding reconstruction statistic as variables are systematically replaced and added to the SMITE pHsw reconstruction. Each line thus ends on the final value of each reconstruction statistic when all seven coral variables are used. (TIF) [file pone.0305607.s003.tif]

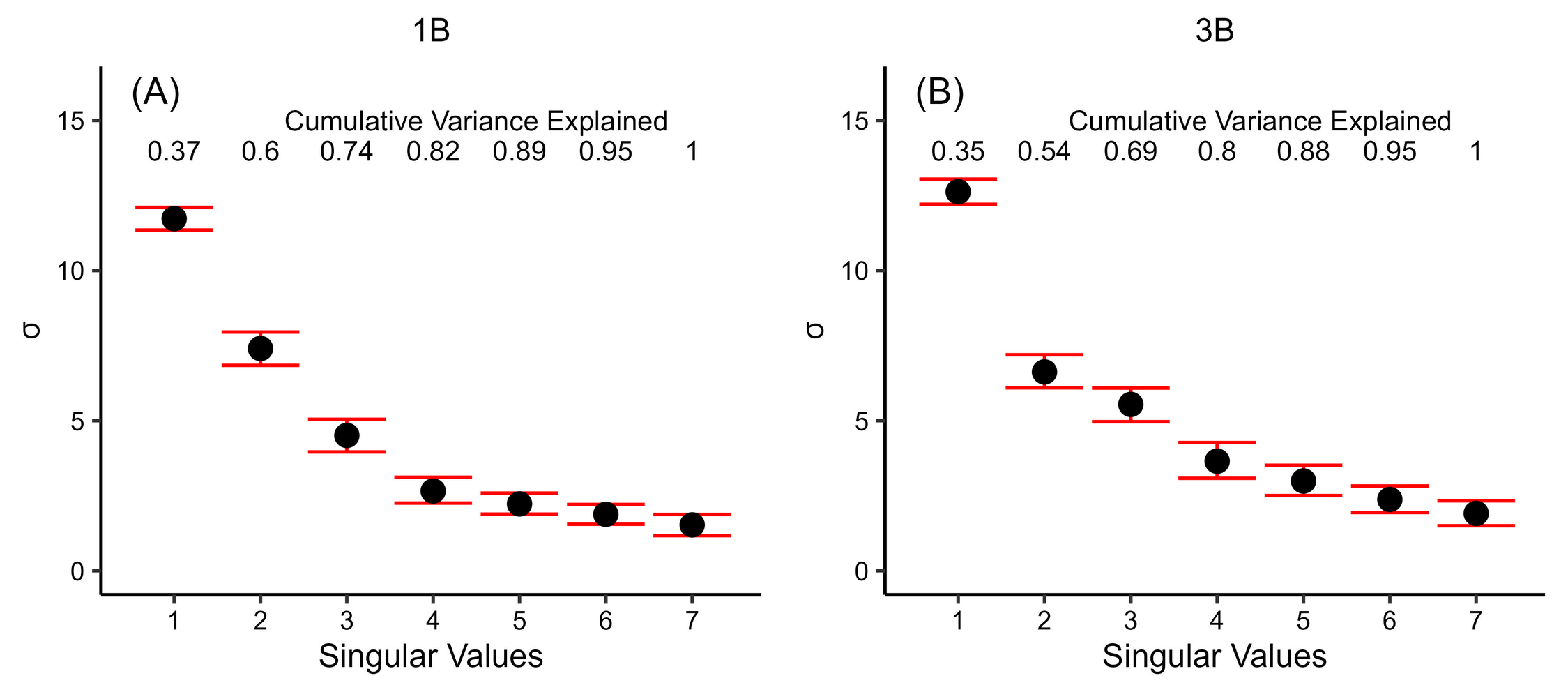

Supplement: S4 Fig — The red error bars around each point indicate the 95% confidence interval estimated using a bootstrap Monte Carlo approach. (TIF) [file pone.0305607.s004.tif]

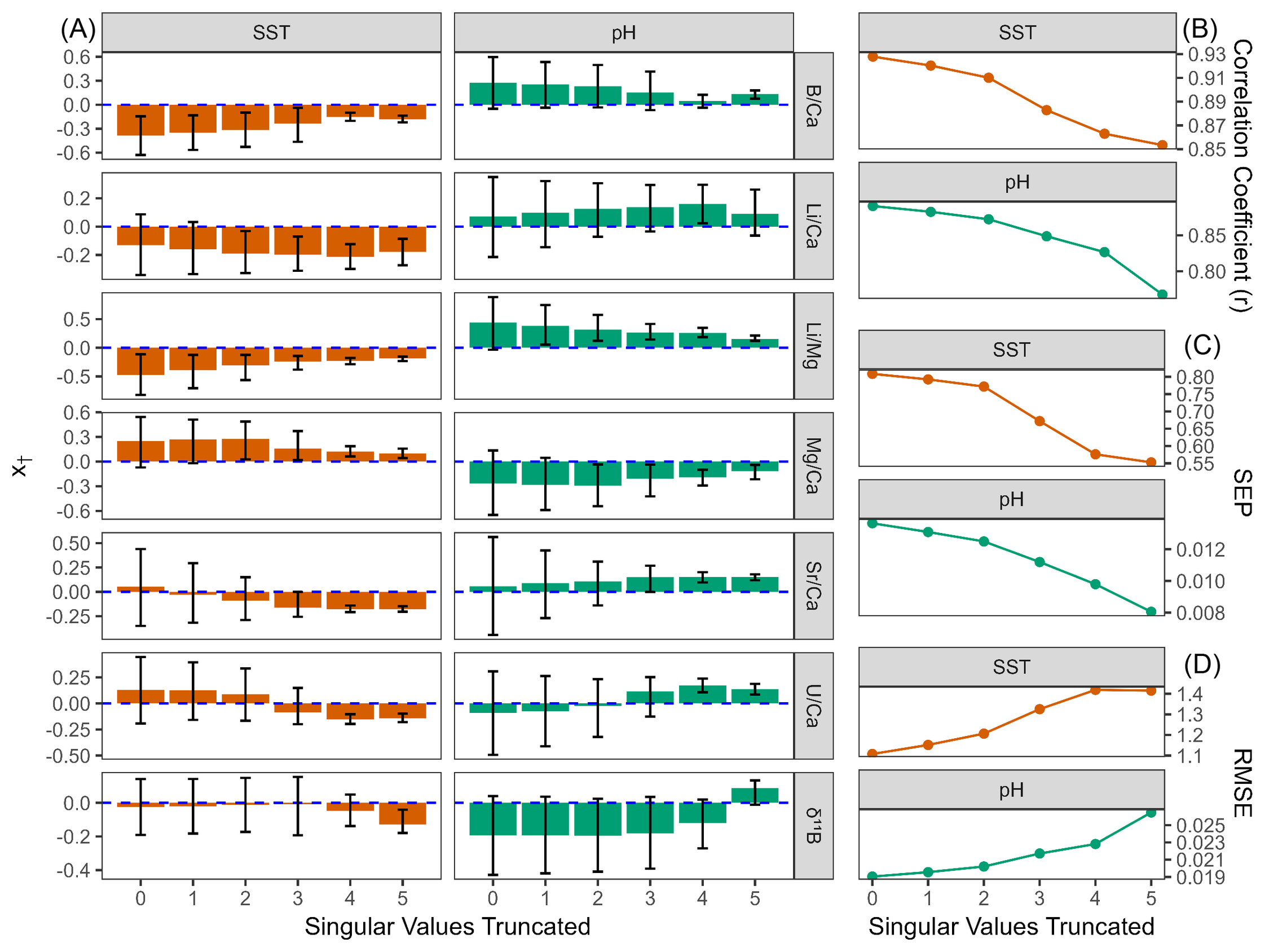

Supplement: S5 Fig — The x-axis in each plot denotes the number of singular values truncated. Each plot thus shows the progressive effects from no truncation (left) to maximum truncation (right). Truncation occurs from the highest (least dominant) singular values to the lowest (most dominant) singular values. The first two singular values can never be truncated. Colors distinguish the results from the SST reconstructions (orange) versus the pHsw reconstructions (green). (A) SMITE model parameters, or x† values, at each successive level of truncation. Rows denote the SMITE model parameter. The colored bar within each plot indicates the x† value of the corresponding SMITE model parameter at a given level of truncation. Error bars for each x† value denote the 95% confidence interval based on a Monte Carlo approach. (B—D) The correlation coefficient (r; B), the standard error of prediction (SEP; C), and the root-mean-square-error (RMSE; D) at each successive level of truncation. (TIF) [file pone.0305607.s005.tif]
