# Supplementary material for: Synthetic and practical reconstructions of SST and seawater pH using the novel multiproxy SMITE method
Source: PLoS One. 2024 Jun 25;19(6):e0305607. doi: 10.1371/journal.pone.0305607 (PMC11198822; doi:10.1371/journal.pone.0305607)
Supplement: S1 Table — (DOCX) [file pone.0305607.s006.docx]

*Table S1. Mean (μ), standard deviation (σ), analytical error (ε), and correlation coefficient (r) to SST and pH_sw_ for each coral variable measured in both Bermudan* P. astreoides *corals (1B and 3B).*

| Coral | 1B | | | | 3B | | | |
| --- | --- | --- | --- | --- | --- | --- | --- | --- |
| Statistic | (μ) | (σ) | (ε) | (r) -  **SST**/pH_sw_ | (μ) | (σ) | (ε) | (r) -  **SST**/ pH_sw_ |
| B/Ca (μmol/mol) | 501.53 | 33.00 | 7.47 | **−0.75** / 0.63 | 501.97 | 25.48 | 8.52 | **−0.77** / 0.63 |
| δ^11^B  (‰) | 23.09 | 0.69 | 0.26 | **−0.41** / 0.33 | 23.17 | 0.57 | 0.23 | **−0.49** / 0.26 |
| Li/Ca (μmol/mol) | 5.54 | 0.26 | 0.10 | **−0.56** / 0.49 | 5.65 | 0.25 | 0.13 | **−0.47** / 0.37 |
| Li/Mg (mmol/mol) | 1.51 | 0.12 | 0.04 | **−0.83** / 0.81 | 1.44 | 0.11 | 0.04 | **−0.85** / 0.83 |
| Mg/Ca (mmol/mol) | 3.78 | 0.27 | 0.12 | **0.57** / −0.58 | 4.01 | 0.27 | 0.13 | **0.70** / −0.72 |
| Sr/Ca (mmol/mol) | 9.06 | 0.12 | 0.04 | **−0.85** / 0.80 | 9.03 | 0.11 | 0.04 | **−0.77** / 0.72 |
| U/Ca (μmol/mol) | 1199.87 | 73.39 | 25.26 | **−0.70** / 0.70 | 1108.87 | 69.95 | 28.13 | **−0.67** / 0.65 |
