# Supplementary material for: Synthetic and practical reconstructions of SST and seawater pH using the novel multiproxy SMITE method
Source: PLoS One. 2024 Jun 25;19(6):e0305607. doi: 10.1371/journal.pone.0305607 (PMC11198822; doi:10.1371/journal.pone.0305607)
Supplement: S2 Table — (DOCX) [file pone.0305607.s007.docx]

*Table S2. Reconstruction statistics for pH_sw_ reconstructions in both Bermudan* P. astreoides *corals (1B and 3B).*

| Coral | 1B | | | 3B | | |
| --- | --- | --- | --- | --- | --- | --- |
| pH_sw_ Reconstruction | r^2^ | SEP | RMSE | r^2^ | SEP | RMSE |
| SMITE | 0.68 | 0.01 | 0.02 | 0.68 | 0.01 | 0.02 |
| δ^11^B | 0.11 | 0.21 | 0.15 | 0.07 | 0.35 | 0.2 |
| Li/Mg | 0.66 | 0.05 | 0.04 | 0.69 | 0.05 | 0.03 |
